# Asymptotically Optimal Status Update Compression in Multi-Source System: Age–Distortion Tradeoff

**DOI:** 10.3390/e27070664

**Published:** 2025-06-20

**Authors:** Jun Li, Wenyi Zhang

**Affiliations:** Department of Electronic Engineering and Information Science, University of Science and Technology of China, Hefei 230026, China; leej@mail.ustc.edu.cn

**Keywords:** age of information, data compression, high-resolution quantization, multi-source system, timely status updates

## Abstract

We consider a compression problem in a multi-source status-updating system through a representative two-source scenario. The status updates are generated by two independent sources following heterogeneous Poisson processes. These updates are then compressed into binary strings and sent to the receiver via a shared, error-free channel with a unit rate. We propose two compression schemes—a multi-quantizer compression scheme, where a dedicated quantizer–encoder pair is assigned to each source for compression, and a single-quantizer compression scheme, employing a unified quantizer–encoder pair shared across both sources. For each scheme, we formulate an optimization problem to jointly design quantizer–encoder pairs, with the objective of minimizing the sum of the average ages subject to a distortion constraint of symbols, respectively. The following three theoretical results are established: (1) The combination of two uniform quantizers with different parameters, along with their corresponding AoI-optimal encoders, provides an asymptotically optimal solution for the multi-quantizer compression scheme. (2) The combination of a piecewise uniform *w*-quantizer with an AoI-optimal encoder provides an asymptotically optimal solution for the single-quantizer compression scheme. (3) For both schemes, the optimal sum of the average ages is asymptotically linear with respect to the log distortion, with the same slope determined by the sources’ arrival rates.

## 1. Introduction

The age of information (AoI) [1]—a metric for data freshness—has attracted significant research interest in diverse areas, including channel coding [2,3,4,5,6], network scheduling [7,8], remote estimation [9,10,11,12], and other related fields. This growing interest stems from the surge of applications demanding timely status updates, such as the Internet of Things (IoT), Vehicular Ad-hoc NETwork (VANET), and surveillance networks [13,14,15]. In practical applications, many systems often deploy multiple nodes to support complex monitoring tasks. Taking the industrial scenario as an example, a sensor network composed of distributed nodes can monitor key indicators such as temperature and pressure in real time, then transmit the data to the central monitoring system through the network in a timely manner. Based on these real-time data streams, factories can further optimize production processes to improve efficiency. However, for analog (i.e., continuous-valued) sources, high-precision encoding typically requires longer transmission times, inevitably causing information staleness at the receiver. Therefore, it is crucial to design an efficient compression scheme to ensure both timely and accurate recovery.

**Figure 1:**
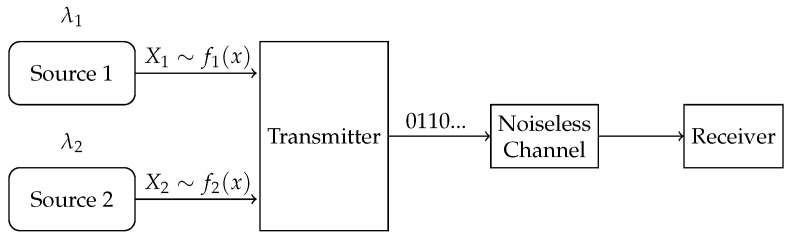
System model.

In this paper, we consider a compression problem in a multi-source (multi-stream) status-updating system through a representative two-source scenario, as illustrated in Figure 1. The system consists of two independent continuous-time analog sources, each generating independent and identically distributed (i.i.d.) symbols with possibly different probability density functions (pdfs). Status updates from the sources arrive according to heterogeneous Poisson processes. These symbols are compressed into binary strings and sent through a shared error-free unit-rate link. When the channel is busy, newly arrived updates are discarded. The system is to be designed to optimize the acquisition of fresh and accurate data at the receiver.

References relevant to this work can be broadly categorized into three research directions.

The first research direction focuses on analyzing the average age in multi-source (multi-stream) status-updating systems. The exact expression of the multi-source M/M/1 non-preemptive queue has been derived in [16]. Furthermore, the average age of the queues with more general service processes has been analyzed. Specifically, Najm and Telatar [17] has investigated the average age for multi-source M/G/1/1 preemptive systems, while Chen et al. [18] has derived the average age for multi-source M/G/1/1 non-preemptive systems, a result particularly relevant to our work. While these works provide fundamental queueing-theoretic insights, they do not incorporate the coding aspects.

The second research direction corresponds to the timely lossless source coding problem under different queuing-theoretic considerations. Therein, transmitting one bit requires one unit of time, so the transmission time of a symbol is equal to the assigned codeword length. Unlike conventional queuing systems with fixed service times, these studies treat codeword lengths as design variables to maximize information freshness, given the symbol arrival processes and probability mass functions (pmf). Existing work has studied various coding schemes under strict lossless reconstruction requirements for the entire data stream—including fixed-to-variable [19], variable-to-variable [20] and variable-to-fixed lossless source coding schemes [21]—as well as more flexible systems permitting symbol skipping when the channel is busy [22,23]. Specifically, Mayekar et al. [22] derived the optimal real-valued codeword lengths under a zero-wait policy, where a new update is generated immediately upon successful delivery of the previous one. In [23], a selective encoding policy was proposed, which discards updates during busy periods and only encodes the most probable *k* realizations; the corresponding optimal real-valued codeword lengths have been also derived. However, these works have only considered single-source scenarios, leaving the multi-source source coding problem unexplored. The analog sources have also not been taken into account, so there is no role of distortion.

The third research direction explores the age–distortion tradeoff, where distortion is defined in different ways across various studies. In [24], distortion was modeled as a monotonically decreasing function of symbol processing time, and the optimal update policies were derived under distortion constraints. In [25], distortion was measured by the mean-squared error (MSE), and the age–distortion tradeoff was studied in a sensing system where the energy harvesting sensor node monitored and transmitted the status updates to the remote monitor. In [26], distortion was modeled as the importance of data, and the age–distortion tradeoff was studied using dynamic programming methods. Another work [27] proposed a cross-layer framework to jointly optimize AoI and compression distortion for real-time monitoring over fading channels. However, these works did not consider the design of variable-rate quantizers.

**Figure 2:**
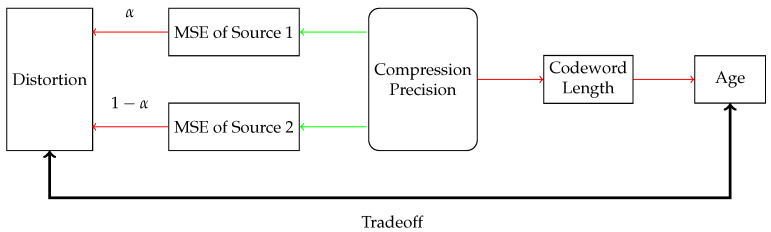
Age–distortion tradeoff schematic diagram in multi-source system. Blocks connected by green (red) arrows represent opposite (same) trend of quantity growth or decrease.

An important case of discrete sources arises from the output of a quantizer. Therefore, we focus on the natural combination of timely lossless source coding and quantization. In our earlier work [28], a joint sampling and compression problem involving the age–distortion tradeoff for a single-source system was investigated, where the arrival process was controlled by the sampler. While [28] established a series of results for single-source systems, multi-source scenarios introduce new issues in the following four aspects: (1) The deployment of multiple quantizers and encoders increases the number of optimization parameters, which are often interdependent. (2) Age evolution in multi-source systems exhibits intrinsic interdependencies distinct from the single-source case. The average age of any individual source may be a nonlinear multivariate function of service times for all sources, which cannot be decoupled, thereby increasing system design complexity. (3) System performance metrics inherently depend on collective behavior across all sources. (4) Significant heterogeneity among sources—in terms of probability distribution characteristics, arrival rates, and other parameters—further complicates the design task. The age–distortion tradeoff visualization is illustrated in Figure 2.

**Figure 3:**
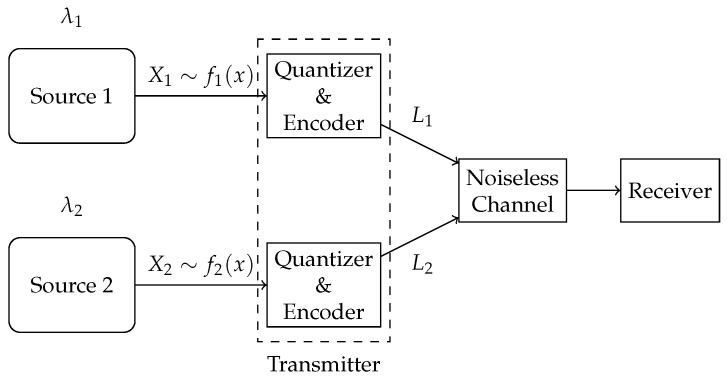
Multi-quantizer compression scheme.

**Figure 4:**
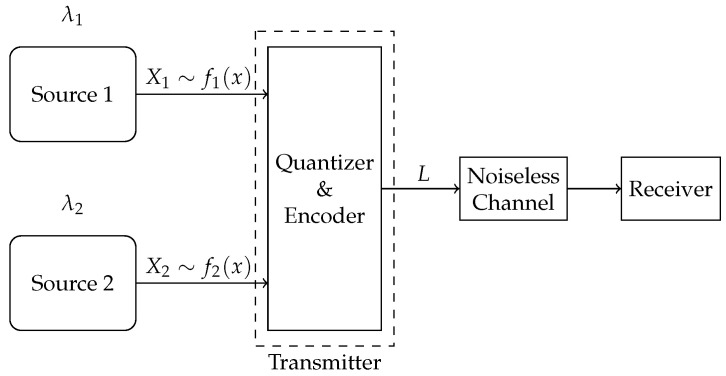
Single-quantizer compression scheme.

To accommodate heterogeneous requirements on accuracy, we introduce weights α and 1−α, and use the weighted sum of mean-squared errors (WSMSE) as the system distortion measure. We propose two compression schemes—a multi-quantizer compression scheme with dedicated quantizer–encoder pairs for each source, and a single-quantizer compression scheme, employing a unified quantizer–encoder pair shared across both sources, as illustrated in Figure 3 and Figure 4, respectively.

For each compression scheme, we formulate a joint optimization problem to design quantizers and encoders, minimizing the sum of the average ages under a given distortion constraint of symbols. For the multi-quantizer compression scheme, the combination of two uniform quantizers with different parameters, along with the corresponding AoI-optimal encoders, provides an asymptotically optimal solution. For the single-quantizer compression scheme, the combination of a piecewise uniform *w*-quantizer with the corresponding AoI-optimal encoder provides an asymptotically optimal solution. Our analysis reveals that the optimal sum of average ages follows an asymptotically linear relationship with log WSMSE, with the same slope determined by the arrival rates of both sources. In comparison, the optimal average age versus log MSE is asymptotically linear with a slope of $-\frac{3}{4}$, as established by [28] for the single-source case. A classical result in the high-resolution quantization theory states that entropy versus log MSE is asymptotically linear with a slope of $-\frac{1}{2}$ [29].

The remainder of this paper is organized as follows: In Section 2, we describe the system model and propose two compression schemes. In Section 3, we study the multi-quantizer compression scheme and develop its asymptotically optimal solution. In Section 4, we turn to the single-quantizer compression scheme and develop its corresponding results. The influence of different parameters on system performance is studied in Section 5. Numerical results are provided in Section 6. Finally, we conclude the paper in Section 7.

## 2. System Model

We consider a continuous-time status-updating system with two independent analog sources, as shown in Figure 1. For each source i,(i=1,2), updating symbols $X_{i}$ are generated as i.i.d. random variables with known pdf $f_{i}\left(x\right)$, and they arrive according to a Poisson process with rate $\lambda_{i}$. We assume that the pdfs satisfy the following conditions—-an assumption that is typically employed in high-resolution quantization theory [30]:

(1) Each pdf $f_{i}\left(x\right)$ is continuous and sufficiently smooth, and both pdfs share the same bounded support interval U=[a,b]. (2) The quantization cells are sufficiently small such that each pdf can be considered approximately constant within each cell. (3) Each reconstruction point $\hat{X}$ is positioned at the centroid of its corresponding quantization cell. (4) The quantization rate *R* is sufficiently high (i.e., R→∞).

We propose two compression schemes—a multi-quantizer compression scheme, where a dedicated quantizer–encoder pair is assigned to each source for compression, and a single-quantizer compression scheme, employing a unified quantizer–encoder pair shared across both sources, as shown in Figure 3 and Figure 4, respectively. The difference between the two schemes lies in their required number of quantizer–encoder pairs. The multi-quantizer compression scheme offers greater design flexibility, as it can be designed separately for each source. In contrast, the single-quantizer compression scheme requires only one quantizer–encoder pair, whose design is dependent on the characteristics of both sources simultaneously, potentially limiting its performance.

During idle channel periods, arriving symbols are quantized and then assigned binary prefix-free codewords by an encoder. These compressed symbols are then sent to the receiver through a shared noiseless channel at a rate of one bit per unit time, while any new arrivals generated during channel busy periods are discarded. Therefore, the transmission time of a symbol is equal to its assigned codeword length, and our system can be modeled as a multi-source (multi-stream) M/G/1/1 non-preemptive system [18]. We assume that the receiver can identify the corresponding source of the symbols received.

We use AoI to quantify information freshness. For source *i*, the AoI at time *t* is defined as $\Delta^{\left(i\right)}\left(t\right)=t-\tau^{\left(i\right)}\left(t\right)$, where $\tau^{\left(i\right)}\left(t\right)$ denotes the generation time of the most recently received update at time *t*, and the average age is given by the following:

(1) $$\begin{array}{c} \Delta^{\left(i\right)}=\lim_{T\to \infty }\frac{1}{T}\int_{0}^{T}\Delta^{\left(i\right)}\left(t\right)dt. \end{array}$$

For the multi-source M/G/1/1 non-preemptive system, the average age of source i,(i=1,2) is given by the following [18]:

(2) $$\begin{array}{c} \Delta^{\left(i\right)}=\frac{\lambda_{1}E\left[S_{1}\right]+\lambda_{2}E\left[S_{2}\right]+1}{\lambda_{i}}+\frac{\lambda_{1}E\left[S_{1}^{2}\right]+\lambda_{2}E\left[S_{2}^{2}\right]}{2(\lambda_{1}E\left[S_{1}\right]+\lambda_{2}E\left[S_{2}\right]+1)}, \end{array}$$

where $S_{1}$ and $S_{2}$ denote the transmission times of symbols generated by sources 1 and 2, respectively.

The sum of the average ages—with the direct use of AoI to represent the sum of the average ages—is expressed as follows:

(3) $$\begin{array}{cc} \Delta = & \frac{\lambda_{1}E\left[S_{1}\right]+\lambda_{2}E\left[S_{2}\right]+1}{\lambda_{1}}+\frac{\lambda_{1}E\left[S_{1}\right]+\lambda_{2}E\left[S_{2}\right]+1}{\lambda_{2}}+\frac{\lambda_{1}E\left[S_{1}^{2}\right]+\lambda_{2}E\left[S_{2}^{2}\right]}{\lambda_{1}E\left[S_{1}\right]+\lambda_{2}E\left[S_{2}\right]+1}. \end{array}$$

### 2.1. Multi-Quantizer Compression Scheme

In the multi-quantizer compression scheme, the symbols from two sources are compressed by two separate quantizer–encoder pairs, respectively. For source *i*, given a quantizer $Q_{i}$, $\left(a_{j_{i}-1},a_{j_{i}}\right]$ is used to denote the $j_{i}$th quantization cell. Each cell is represented by a reproduction point $c_{j_{i}}$ with occurrence probability $p_{j_{i}}$. Let Q denote the set of quantizers. The MSE for source *i* is expressed as follows:

(4) $$\begin{array}{c} D\left(Q_{i}\left(X_{i}\right)\right)=\sum_{j_{i}}\int_{a_{j_{i}-1}}^{a_{j_{i}}}{\left(x-c_{j_{i}}\right)}^{2}f_{i}\left(x\right)dx. \end{array}$$

To accommodate heterogeneous requirements on accuracy, we introduce weights α and 1−α. For the multi-quantizer compression scheme, we define the WSMSE $D_{m}\left(X_{1},X_{2}\right)$ as the system distortion measure, as expressed in Equation (5). In this paper, we use the subscript “m” to denote variables under the multi-quantizer compression scheme, and “s” for variables under the single-quantizer compression scheme.

(5) $$\begin{array}{c} D_{m}\left(X_{1},X_{2}\right)=\alpha D\left(Q_{1}\left(X_{1}\right)\right)+\left(1-\alpha \right)D\left(Q_{2}\left(X_{2}\right)\right). \end{array}$$

For a given pmf, we assign binary prefix-free codewords to the quantization cells, where the codeword length for the *j*th cell of source *i* is denoted by $l_{j_{i}}$. The random variable $L_{i}$ represents the codeword length for the quantized symbols of source *i*, and the set of all prefix-free codeword length assignments is denoted by L. As established in information theory, the codeword lengths of any prefix-free code must satisfy the Kraft inequality, i.e., $\sum_{j_{i}}2^{-l_{j_{i}}}\leq 1$ ([29], p. 24). In our analysis, we focus on the high-resolution regime where the average codeword lengths are sufficiently large. In subsequent analysis, we ignore the integer constraint and consider the real-valued length assignments. The transmission time of a symbol is equal to its assigned codeword length; the AoI is given by the following:

(6) $$\begin{array}{cc} \Delta_{m}= & \frac{\lambda_{1}E\left[L_{1}\right]+\lambda_{2}E\left[L_{2}\right]+1}{\lambda_{1}}+\frac{\lambda_{1}E\left[L_{1}\right]+\lambda_{2}E\left[L_{2}\right]+1}{\lambda_{2}}+\frac{\lambda_{1}E\left[L_{1}^{2}\right]+\lambda_{2}E\left[L_{2}^{2}\right]}{\lambda_{1}E\left[L_{1}\right]+\lambda_{2}E\left[L_{2}\right]+1}. \end{array}$$

The AoI $\Delta_{m}$ is a nonlinear function of $E[L_{i}]$ and $E[L_{i}^{2}]$, which is difficult to decouple directly. Furthermore, each quantizer $Q_{i}$ determines the output pmf $P_{i}\left(Q_{i}\left(X_{i}\right)\right)$. Therefore, the AoI minimization problem can be formulated as a joint codeword length assignment problem, which is a complex nonlinear fractional problem that involves the joint design of two sets of codeword lengths, as follows:

(7) $$\begin{array}{cc} \; & \min_{\left\{L_{1},L_{2}\in L\right\}}\Delta_{m}\\ s.t. & \sum_{j_{1}}2^{-l_{j_{1}}}\leq 1,\;\sum_{j_{2}}2^{-l_{j_{2}}}\leq 1,\;l_{j_{1}},l_{j_{2}}\in R^{+}. \end{array}$$

Given the arrival processes for both sources, the AoI is governed by the assigned codeword lengths, which in turn are determined by the pmfs of the quantizer outputs. The WSMSE distortion metric, characterized by the MSEs for both sources, is directly influenced by the design of both quantizers. When the analog sources are considered, this framework naturally gives rise to an inherent tradeoff between AoI and distortion performance, which necessitates the joint optimization of both quantizer-encoder pairs, leading to a generalized formulation of Problem (7) as follows:

(8) $$\begin{array}{cc} \; & \min_{\left\{Q_{1},Q_{2}\in Q,L_{1},L_{2}\in L\right\}}\Delta_{m}\\ s.t. & \sum_{j_{1}}2^{-l_{j_{1}}}\leq 1,\;\sum_{j_{2}}2^{-l_{j_{2}}}\leq 1,\;D_{m}\left(X_{1},X_{2}\right)\leq D,\;l_{j_{1}},l_{j_{2}}\in R^{+}. \end{array}$$

### 2.2. Single-Quantizer Compression Scheme

In the single-quantizer compression scheme, the symbols generated by both sources are compressed by a shared quantizer–encoder pair. Therefore, the design of this pair depends simultaneously on both sources.

For a given quantizer *Q*, we denote the *j*th quantization cell by $\left(a_{j-1},a_{j}\right]$, represented by a reproduction point $c_{j}$. The quantizer maps continuous inputs $X_{1}$ and $X_{2}$ to discrete outputs $Q(X_{1})$ and $Q(X_{2})$, with pmfs $P_{1}\left(Q\left(X_{1}\right)\right)=\left\{p_{1},p_{2},\ldots ,p_{j},\ldots \right\}$ and $P_{2}\left(Q\left(X_{2}\right)\right)=\left\{q_{1},q_{2},\ldots ,q_{j},\ldots \right\}$, defined as follows:

(9) $$\begin{array}{c} p_{j}=\int_{a_{j-1}}^{a_{j}}f_{1}\left(x\right)dx,\;q_{j}=\int_{a_{j-1}}^{a_{j}}f_{2}\left(x\right)dx \end{array}$$

representing the occurrence probabilities for sources 1 and 2, respectively. The MSE for source *i* is given by the following:

(10) $$\begin{array}{c} D\left(Q\left(X_{i}\right)\right)=\sum_{j}\int_{a_{j-1}}^{a_{j}}{\left(x-c_{j}\right)}^{2}f_{i}\left(x\right)dx. \end{array}$$

For the single-quantizer compression scheme, we similarly adopt the WSMSE $D_{s}\left(X_{1},X_{2}\right)$ as system distortion metric, as follows:

(11) $$\begin{array}{c} D_{s}\left(X_{1},X_{2}\right)=\alpha D\left(Q\left(X_{1}\right)\right)+\left(1-\alpha \right)D\left(Q\left(X_{2}\right)\right). \end{array}$$

We assign binary prefix-free codewords to the quantization cells, where the codeword length assigned to the *j*th cell is denoted by $l_{j}$. Since both sources share the same quantizer–encoder pair, the quantization regions, reproduction points, and codeword length assignments remain identical. However, the pmfs of the codeword length assignments are different, leading to distinct statistical properties of the transmission times. Although the same symbol has identical transmission time values for both sources, their distributions differ. To explicitly capture the differences in distribution, we denote the first and second moments of the codeword lengths as $E_{P_{i}}\left[L\right]$ and $E_{P_{i}}\left[L^{2}\right]$ for each source *i*, respectively. The AoI is given by

(12) $$\begin{array}{c} \Delta_{s}=\frac{\lambda_{1}E_{P_{1}}\left[L\right]+\lambda_{2}E_{P_{2}}\left[L\right]+1}{\lambda_{1}}+\frac{\lambda_{1}E_{P_{1}}\left[L\right]+\lambda_{2}E_{P_{2}}\left[L\right]+1}{\lambda_{2}}+\frac{\lambda_{1}E_{P_{1}}\left[L^{2}\right]+\lambda_{2}E_{P_{2}}\left[L^{2}\right]}{\lambda_{1}E_{P_{1}}\left[L\right]+\lambda_{2}E_{P_{2}}\left[L\right]+1}. \end{array}$$

The AoI $\Delta_{s}$ is a nonlinear function of $E_{P_{i}}\left[L\right]$ and $E_{P_{i}}\left[L^{2}\right]$, which is difficult to decouple directly. Furthermore, the quantizer *Q* determines the output pmfs $P_{i}\left(Q\left(X_{i}\right)\right)$ for source *i*, thereby necessitating the design of codeword lengths for both pmfs. This leads to the following AoI minimization problem:

(13) $$\begin{array}{cc} \; & \min_{\left\{L\in L\right\}}\Delta_{s}\\ s.t. & \sum_{j}2^{-l_{j}}\leq 1,\;l_{j}\in R^{+}. \end{array}$$

When the analog sources are considered, this framework naturally gives rise to an inherent tradeoff between AoI and distortion performance. Specifically, for both sources, the quantizer design and codeword length assignment are intrinsically coupled. The quantizer determines both the distortion characteristics and the output probability distributions, which subsequently govern the optimal codeword length assignment and, consequently, the achievable AoI performance. We study a joint quantization and encoding problem to optimize AoI under a distortion constraint, as follows:

(14) $$\begin{array}{cc} \; & \min_{\left\{Q\in Q,L\in L\right\}}\Delta_{s}\\ s.t. & \sum_{j}2^{-l_{j}}\leq 1,\;D_{s}\left(X_{1},X_{2}\right)\leq D,\;l_{j}\in R^{+}. \end{array}$$

The encoders are different between the two compression schemes. The multi-quantizer scheme employs two distinct sets of codeword lengths (one for each source), whereas the single-quantizer scheme employs a unified set of codeword lengths for both sources. To maintain notational simplicity, for both schemes, we refer to encoders with codeword lengths satisfying optimization problems (7) and (13) collectively as the AoI-optimal encoder, denoted by $F^{*}$. In addition, the Shannon encoder $F_{s}$ is defined as the encoder with lengths $l_{j}=-\log_{2}p_{j}$, where $p_{j}$ represents the probability of the *j*th realization.

### 2.3. Preliminaries

In this section, we present some definitions that will be used throughout the rest of the text.

**Definition 1.**  *For the multi-quantizer compression scheme, given a distortion threshold D>0, the optimal AoI is defined as follows:*

(15) Δm*(D)=infQ1,Q2,F,Dm(X1,X2)≤DΔm(Q1,Q2,F),

*where the infimum is taken over all quantizers $Q_{1},Q_{2}$ satisfying the distortion constraint and codeword length assignments $L_{1},L_{2}$.*

*A quintuple $\left(Q_{1}^{\prime },Q_{2}^{\prime },F^{\prime },D_{1},D_{2}\right)$ is asymptotically optimal under the distortion threshold D if:*

(16) $$\begin{array}{c} \lim_{D\to 0}\left[\Delta_{m}\left(Q_{1}^{\prime },Q_{2}^{\prime },F^{\prime },D_{1},D_{2}\right)-\Delta_{m}^{*}\left(D\right)\right]=0. \end{array}$$

*For any distortion thresholds $D_{1}$ and $D_{2}$ satisfying $\alpha D_{1}+\left(1-\alpha \right)D_{2}\leq D$, we define the constrained optimal AoI under the distortion thresholds $D_{1}$ and $D_{2}$ as follows:*

(17) Δm*(D1,D2)=infQ1,Q2,F,D(Q1(X1))≤D1,D(Q2(X2))≤D2Δm(Q1,Q2,F).

*Similarly, a triplet $\left(Q_{1}^{\prime },Q_{2}^{\prime },F^{\prime }\right)$ is asymptotically optimal under the distortion thresholds $D_{1}$ and $D_{2}$ if:*

(18) limD1→0D2→0[Δm(Q1′,Q2′,F′)−Δm*(D1,D2)]=0.

**Remark 1.**  *Since the system distortion metric is the WSMSE, in the multi-quantizer compression scheme and under a distortion threshold D>0, the design problem not only involves the design of the quantizer and corresponding encoders but also needs to allocate the appropriate distortion for each source. Consequently, the complete solution can be formally represented as a quintuple $\left(Q_{1},Q_{2},F,D_{1},D_{2}\right)$.*

**Definition 2.**  *For the single-quantizer compression scheme, given a distortion threshold D>0, the optimal AoI is given by the following:*

(19) Δs*(D)=infQ,F,Ds(X1,X2)≤DΔs(Q,F).

*A pair $\left(Q^{\prime },F^{\prime }\right)$ is asymptotically optimal under the distortion threshold D if:*

(20) $$\begin{array}{c} \lim_{D\to 0}\left[\Delta_{s}\left(Q^{\prime },F^{\prime }\right)-\Delta_{s}^{*}\left(D\right)\right]=0. \end{array}$$

Subsequently, we present some definitions and known results of the high-resolution quantization theory. Given a quantizer *Q*, the output entropy is denoted by H(Q(X)). A quantizer that achieves the minimum entropy for a given distortion threshold *D* is called the optimal quantizer denoted by $Q^{*}$. The uniform quantizer is denoted by $Q_{\mathrm{uni}}$. The definition of an asymptotically optimal quantizer is as follows:

**Definition 3.**  *A quantizer Q is asymptotically optimal if*

(21) $$\begin{array}{c} \lim_{D\to 0}\left[H\left(Q\left(X\right)\right)-H\left(Q^{*}\left(X\right)\right)\right]=0. \end{array}$$

For a pdf f(x) and a weight function w(x), the weighted mean-squared error (WMSE) distortion is defined by the following:

(22) $$\begin{array}{c} D_{w}\left(Q\left(X\right)\right)=\sum_{j}\int_{a_{j-1}}^{a_{j}}w\left(x\right){\left(x-c_{j}\right)}^{2}f\left(x\right)dx. \end{array}$$

According to the results of [30], for a continuous and sufficiently smooth weight function w(x) and the corresponding WMSE distortion, a piecewise uniform quantizer—which we call the *w*-quantizer denoted by $Q_{w}$—can be constructed. The construction proceeds as follows: First, the support interval *U* is partitioned into intervals $\left\{I_{1},\ldots ,I_{j},\ldots \right\}$ of equal step size δ. For sufficiently small δ, w(x) is approximately constant $w(x_{j})$ within each $I_{j}$. Then, each interval $I_{j}$ is subdivided into the cells with length $\frac{\delta }{\sqrt{w(x_{j})}}$, and the midpoint of each cell is the reproduction point. The results are recapitulated as follows:

**Lemma 1.**  ([30]). *Let w(x) be a continuous, sufficiently smooth weight function with the bounded support interval U=[a,b]. Under the WMSE distortion, the w-quantizer is asymptotically optimal, as follows:*

(23) $$\begin{array}{c} \lim_{D\to 0}H\left[\left(Q_{w}\left(X\right)\right)-H\left(Q^{*}\left(X\right)\right)\right]=0. \end{array}$$

*Furthermore, the asymptotic behavior of the distortion satisfies the following:*

(24) $$\begin{array}{c} \lim_{\delta \to 0}\frac{D}{\delta^{2}}=\frac{1}{12}. \end{array}$$

*and*

(25) $$\begin{array}{c} \lim_{D\to 0}\left[H\left(Q_{w}\left(X\right)\right)+\log_{2}\sqrt{12D}\right]=h\left(X\right)+\frac{1}{2}E\left[\log_{2}\left(w\left(X\right)\right)\right], \end{array}$$

*where*

(26) $$\begin{array}{c} h\left(X\right)=-\int_{U}f\left(x\right)\log_{2}f\left(x\right)dx \end{array}$$

*is the differential entropy of the random variable X.*

**Remark 2.**  *A well-known result in the high-resolution quantization theory is that the uniform quantizer is asymptotically optimal for entropy-constrained quantization [31], which is a special case of w(x)≡1. The key properties follow from Lemma 1 with w(x)=1 directly, as follows:*

(27) $$\begin{array}{c} \lim_{D\to 0}\left[H\left(Q_{\mathrm{uni}}\left(X\right)\right)+\log_{2}\sqrt{12D}\right]=h\left(X\right). \end{array}$$

## 3. Asymptotically Optimal Solution for Multi-Quantizer Compression Scheme

For the multi-quantizer compression scheme, we develop an asymptotically optimal solution. For notational simplicity, we define the following for use throughout the paper:

(28) $$\begin{array}{cc} \; & a:=\frac{\lambda_{1}}{\lambda_{2}}+\frac{\lambda_{1}}{\lambda_{1}+\lambda_{2}}+1 \end{array}$$

(29) $$\begin{array}{cc} \; & b:=\frac{\lambda_{2}}{\lambda_{1}}+\frac{\lambda_{2}}{\lambda_{1}+\lambda_{2}}+1 \end{array}$$

(30) $$\begin{array}{cc} \; & c:=\frac{\lambda_{1}^{2}+\lambda_{1}\lambda_{2}+\lambda_{2}^{2}}{\lambda_{1}\lambda_{2}\left(\lambda_{1}+\lambda_{2}\right)} \end{array}$$

The results are then presented as follows:

**Theorem 1.**  The uniform quantizer $Q_{\mathrm{uni}}^{\left(1\right)}$, with cell size $\delta_{1}=\sqrt{\frac{12aD}{\alpha (a+b)}}$ and distortion $D_{1}^{*}=\frac{aD}{\alpha (a+b)}$ for source 1, along with the uniform quantizer $Q_{\mathrm{uni}}^{\left(2\right)}$ with cell size $\delta_{2}=\sqrt{\frac{12bD}{\left(1-\alpha )(a+b\right)}}$ and distortion $D_{2}^{*}=\frac{bD}{\left(1-\alpha )(a+b\right)}$ for source 2, as well as the AoI-optimal encoder, together provide an asymptotically optimal solution to the problem (8), in the sense that, as the distortion D→0, this solution asymptotically achieves the optimal AoI.

(31) $$\begin{array}{c} \lim_{D\to 0}\left[\Delta_{m}\left(Q_{\mathrm{uni}}^{\left(1\right)},Q_{\mathrm{uni}}^{\left(2\right)},F^{*},D_{1}^{*},D_{2}^{*}\right)-\Delta_{m}^{*}\left(D\right)\right]=0. \end{array}$$

*Under this solution, the asymptotic behavior of the optimal AoI satisfies the following:*

(32) $$\begin{array}{cc} \lim_{D\to 0} & \left[\Delta_{m}^{*}\left(D\right)+\frac{a+b}{2}\log_{2}12D\right]\\ \; & \;=-\frac{a}{2}\log_{2}\frac{a}{\alpha (a+b)}-\frac{b}{2}\log_{2}\frac{b}{\left(1-\alpha )(a+b\right)}+ah\left(X_{1}\right)+bh\left(X_{2}\right)+c \end{array}$$

*and*

(33) $$\begin{array}{c} \lim_{D\to 0}\frac{\Delta_{m}^{*}\left(D\right)}{\log_{2}D}=-\frac{a+b}{2}. \end{array}$$

**Remark 3.**  Theorem 1 reveals that the performance of the multi-quantizer compression scheme exhibits a strong dependence on the weight α and the ratio $\frac{a}{a+b}$, since the allocation of distortion for each source is directly determined by these parameters. The ratio $\frac{a}{a+b}$ quantifies the relative contribution of source 1 to the sum of the average ages, as implied in the proof below. Intuitively, a larger ratio $\frac{a}{a+b}$ necessitates a smaller average age for source 1 to minimize the sum of the average ages, and a smaller value of α indicates that the source can tolerate a larger distortion. This is consistent with the optimal distortion allocation $D_{1}^{*}=\frac{aD}{\alpha (a+b)}$. The analysis for $D_{2}^{*}$ follows analogously.

**Remark 4.**  *Let*

(34) $$\begin{array}{c} \delta =\sqrt{\frac{\alpha (a+b)}{a}}\delta_{1}=\sqrt{\frac{\left(1-\alpha )(a+b\right)}{b}}\delta_{2}. \end{array}$$

As $\delta_{1}\to 0$ and $\delta_{2}\to 0$, $D_{1}^{*}\to \frac{\delta_{1}^{2}}{12}$ and $D_{2}^{*}\to \frac{\delta_{2}^{2}}{12}$. Then,

(35) $$\begin{array}{c} \alpha \frac{\delta_{1}^{2}}{12}+\left(1-\alpha \right)\frac{\delta_{2}^{2}}{12}=\alpha \frac{a}{\alpha (a+b)}\frac{\delta^{2}}{12}+\left(1-\alpha \right)\frac{b}{\left(1-\alpha )(a+b\right)}\frac{\delta^{2}}{12}=\frac{\delta^{2}}{12}, \end{array}$$

*which yields*

(36) $$\begin{array}{c} D=\alpha D_{1}^{*}+\left(1-\alpha \right)D_{2}^{*}\to \frac{\delta^{2}}{12}. \end{array}$$

*Thus, we define δ as the step size for the multi-quantizer compression scheme.*

**Remark 5.**  *A classical result in the high-resolution quantization theory is that entropy versus log MSE is asymptotically linear with a slope of $-\frac{1}{2}$ [29]. Similarly, our multi-quantizer scheme reveals an analogous asymptotically linear relationship—the performance curve of the optimal AoI versus log WSMSE is asymptotically linear with a slope of $-\frac{a+b}{2}$, which depends explicitly on the source arrival rates.*

**Figure 5:**
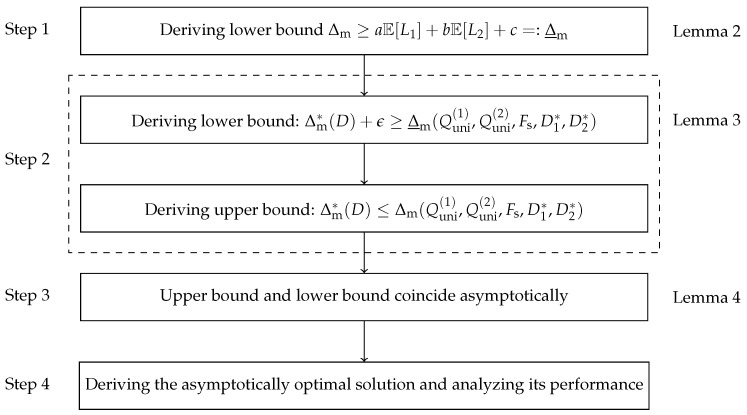
Proof flowchart of Theorem 1.

The proof of Theorem 1 proceeds as follows: First, a lower bound of the AoI $\Delta_{m}$ is constructed, which decouples the design of codeword length assignments for both sources. Then, we obtain the upper and asymptotically lower bounds of the optimal AoI $\Delta_{m}^{*}\left(D\right)$ through (i) allocating appropriate distortion to each source and (ii) designing the corresponding quantizer–encoder pair. We further prove that the two bounds are tight asymptotically. During this process, to avoid directly solving Problem (8), the performance of the Shannon encoder is used to approximate the solution of (8) in the high-resolution regime. Finally, we prove the asymptotically optimality of the solution and analyze its performance. The proof flowchart of Theorem 1 is shown in Figure 5.

**Proof.**  We divide the proof of Theorem 1 into four steps.

**Step 1.** Derive the lower bound of $\Delta_{m}$, as follows:

**Lemma 2.**  *$\Delta_{m}$ is lower bounded by the following:*

(37) $$\begin{array}{cc} \; & \Delta_{m}\geq aE\left[L_{1}\right]+bE\left[L_{2}\right]+c=:{\underline{\Delta }}_{m}. \end{array}$$

**Proof.**  See Appendix A. □

**Step 2.** Derive the upper and asymptotically lower bounds of the optimal AoI $\Delta_{m}^{*}\left(D\right)$.

For notational simplicity, we define the following:

(38) $$\begin{array}{c} {\underline{\Delta }}_{m}\left(Q_{\mathrm{uni}}^{\left(1\right)},Q_{\mathrm{uni}}^{\left(2\right)},F_{s},D_{1},D_{2}\right):=aH\left(Q_{\mathrm{uni}}^{\left(1\right)}\left(X_{1}\right),D_{1}\right)+bH\left(Q_{\mathrm{uni}}^{\left(2\right)}\left(X_{2}\right),D_{2}\right)+c, \end{array}$$

where $H(Q_{\mathrm{uni}}^{\left(i\right)}\left(X_{i}\right),D_{i})$ denotes the output entropy of the uniform quantizer for source i,(i=1,2) with the allocated distortion of $D_{i}$.

In the following lemma, an asymptotically lower bound of $\Delta_{m}^{*}\left(D\right)$ is given:

**Lemma 3.**  *For any ϵ>0, there exists a distortion $D_{0}>0$ such that $0<D<D_{0}$, the following inequality holds:*

(39) $$\begin{array}{cc} \Delta_{m}^{*}\left(D\right)+\epsilon & \geq {\underline{\Delta }}_{m}\left(Q_{\mathrm{uni}}^{\left(1\right)},Q_{\mathrm{uni}}^{\left(2\right)},F_{s},D_{1}^{*},D_{2}^{*}\right), \end{array}$$

where $D_{1}^{*}=\frac{aD}{\alpha (a+b)}$ and $D_{2}^{*}=\frac{bD}{\left(1-\alpha )(a+b\right)}$.

**Proof.**  See Appendix B. □

Given the optimal distortion allocation scheme $\left(D_{1}^{*},D_{2}^{*}\right)$, the AoI achieved by the corresponding uniform quantizers with the Shannon encoders $\Delta_{m}\left(Q_{\mathrm{uni}}^{\left(1\right)},Q_{\mathrm{uni}}^{\left(2\right)},F_{s},D_{1}^{*},D_{2}^{*}\right)$ is obviously an upper bound of the optimal AoI, expressed as follows:

(40) $$\begin{array}{c} \Delta_{m}^{*}\left(D\right)\leq \Delta_{m}\left(Q_{\mathrm{uni}}^{\left(1\right)},Q_{\mathrm{uni}}^{\left(2\right)},F_{s},D_{1}^{*},D_{2}^{*}\right). \end{array}$$

**Step 3.** We proceed to prove that the upper bound and the asymptotically lower bound of the optimal AoI coincide asymptotically.

**Lemma 4.**  *If the uniform quantizers and the Shannon encoders for sources 1 and 2 are given, then*

(41) $$\begin{array}{c} \lim_{D\to 0}\left[\Delta_{m}\left(Q_{\mathrm{uni}}^{\left(1\right)},Q_{\mathrm{uni}}^{\left(2\right)},F_{s},D_{1}^{*},D_{2}^{*}\right)-{\underline{\Delta }}_{m}\left(Q_{\mathrm{uni}}^{\left(1\right)},Q_{\mathrm{uni}}^{\left(2\right)},F_{s},D_{1}^{*},D_{2}^{*}\right)\right]=0, \end{array}$$

where $D_{1}^{*}=\frac{aD}{\alpha (a+b)}$ and $D_{2}^{*}=\frac{bD}{\left(1-\alpha )(a+b\right)}$.

**Proof.**  See Appendix C. □

**Step 4.** Derive the asymptotically optimal solution and analyze its performance.

From Lemma 4, we can obtain that, for any ϵ>0, there exists some $D_{0}^{\prime }>0$, such that $0<D<D_{0}^{\prime }$ implies the following:

(42) $$\begin{array}{c} |\Delta_{m}\left(Q_{\mathrm{uni}}^{\left(1\right)},Q_{\mathrm{uni}}^{\left(2\right)},F_{s},D_{1}^{*},D_{2}^{*}\right)-{\underline{\Delta }}_{m}\left(Q_{\mathrm{uni}}^{\left(1\right)},Q_{\mathrm{uni}}^{\left(2\right)},F_{s},D_{1}^{*},D_{2}^{*}\right)|<\frac{\epsilon }{2}. \end{array}$$

Letting $D_{0}^{\prime \prime }=\min\left\{D_{0},D_{0}^{\prime }\right\}$, for any $D_{1}$, $D_{2}$ satisfying $0<D_{1}<D_{0}^{\prime \prime }$ and $0<D_{2}<D_{0}^{\prime \prime }$, the following inequality holds:

(43) $$\begin{array}{c} \Delta_{m}^{*}\left(D\right)+\frac{3}{2}\epsilon >{\underline{\Delta }}_{m}\left(Q_{\mathrm{uni}}^{\left(1\right)},Q_{\mathrm{uni}}^{\left(2\right)},F_{s},D_{1}^{*},D_{2}^{*}\right)+\frac{\epsilon }{2}>\Delta_{m}\left(Q_{\mathrm{uni}}^{\left(1\right)},Q_{\mathrm{uni}}^{\left(2\right)},F_{s},D_{1}^{*},D_{2}^{*}\right). \end{array}$$

Since ϵ can be arbitrarily small, for sufficiently small *D*, the following inequality holds:

(44) $$\begin{array}{c} \Delta_{m}^{*}\left(D\right)\geq \Delta_{m}\left(Q_{\mathrm{uni}}^{\left(1\right)},Q_{\mathrm{uni}}^{\left(2\right)},F_{s},D_{1}^{*},D_{2}^{*}\right)\geq \Delta_{m}\left(Q_{\mathrm{uni}}^{\left(1\right)},Q_{\mathrm{uni}}^{\left(2\right)},F^{*},D_{1}^{*},D_{2}^{*}\right). \end{array}$$

Thus, the quintuple $\left(Q_{\mathrm{uni}}^{\left(1\right)},Q_{\mathrm{uni}}^{\left(2\right)},F^{*},D_{1}^{*},D_{2}^{*}\right)$ is the asymptotically optimal solution.

Next, we prove (32) and (33). We have the following:

(45) $$\begin{array}{cc} \; & |\Delta_{m}^{*}\left(D\right)+\frac{a+b}{2}\log_{2}12D\\ \; & \;+\frac{a}{2}\log_{2}\frac{a}{\alpha (a+b)}+\frac{b}{2}\log_{2}\frac{b}{\left(1-\alpha )(a+b\right)}-ah\left(X_{1}\right)-bh\left(X_{2}\right)-c|\\ \leq & \left|\Delta_{m}\left(Q_{\mathrm{uni}}^{\left(1\right)},Q_{\mathrm{uni}}^{\left(2\right)},F_{s},D_{1}^{*},D_{2}^{*}\right)-{\underline{\Delta }}_{m}\left(Q_{\mathrm{uni}}^{\left(1\right)},Q_{\mathrm{uni}}^{\left(2\right)},F_{s},D_{1}^{*},D_{2}^{*}\right)\right|\\ \; & +|{\underline{\Delta }}_{m}\left(Q_{\mathrm{uni}}^{\left(1\right)},Q_{\mathrm{uni}}^{\left(2\right)},F_{s},D_{1}^{*},D_{2}^{*}\right)+\frac{a+b}{2}\log_{2}12D\\ \; & \;+\frac{a}{2}\log_{2}\frac{a}{\alpha (a+b)}+\frac{b}{2}\log_{2}\frac{b}{\left(1-\alpha )(a+b\right)}-ah\left(X_{1}\right)-bh\left(X_{2}\right)-c|. \end{array}$$

Then, we derive the following:

(46) $$\begin{array}{cc} \; & {\underline{\Delta }}_{m}\left(Q_{\mathrm{uni}}^{\left(1\right)},Q_{\mathrm{uni}}^{\left(2\right)},F_{s},D_{1}^{*},D_{2}^{*}\right)+\frac{a+b}{2}\log_{2}12D\\ \; & +\frac{a}{2}\log_{2}\frac{a}{\alpha (a+b)}+\frac{b}{2}\log_{2}\frac{b}{\left(1-\alpha )(a+b\right)}-ah\left(X_{1}\right)-bh\left(X_{2}\right)-c\\ = & aH\left(Q_{\mathrm{uni}}^{\left(1\right)}\left(X_{1}\right),D_{1}^{*}\right)+bH\left(Q_{\mathrm{uni}}^{\left(2\right)}\left(X_{2}\right),D_{2}^{*}\right)+c+\frac{a+b}{2}\log_{2}12D\\ \; & +\frac{a}{2}\log_{2}\frac{a}{\alpha (a+b)}+\frac{b}{2}\log_{2}\frac{b}{\left(1-\alpha )(a+b\right)}-ah\left(X_{1}\right)-bh\left(X_{2}\right)-c\\ = & aH\left(Q_{\mathrm{uni}}^{\left(1\right)}\left(X_{1}\right),D_{1}^{*}\right)+\frac{a}{2}\log_{2}\frac{12aD}{\alpha (a+b)}-ah\left(X_{1}\right)\\ \; & +bH\left(Q_{\mathrm{uni}}^{\left(2\right)}\left(X_{2}\right),D_{2}^{*}\right)+\frac{b}{2}\log_{2}\frac{12bD}{\left(1-\alpha )(a+b\right)}-bh\left(X_{2}\right)\\ = & aH\left(Q_{\mathrm{uni}}^{\left(1\right)}\left(X_{1}\right),D_{1}^{*}\right)+a\log_{2}\sqrt{12D_{1}^{*}}-ah\left(X_{1}\right)\\ \; & +bH\left(Q_{\mathrm{uni}}^{\left(2\right)}\left(X_{2}\right),D_{2}^{*}\right)+b\log_{2}\sqrt{12D_{2}^{*}}-bh\left(X_{2}\right). \end{array}$$

From the results of the high-resolution theory, then we obtain the following:

(47) $$\begin{array}{c} \lim_{D_{1}^{*}\to 0}\left[aH\left(Q_{\mathrm{uni}}^{\left(1\right)}\left(X_{1}\right),D_{1}^{*}\right)+a\log_{2}\sqrt{12D_{1}^{*}}\;\right]=ah\left(X_{1}\right) \end{array}$$

(48) $$\begin{array}{c} \lim_{D_{2}^{*}\to 0}\left[bH\left(Q_{\mathrm{uni}}^{\left(2\right)}\left(X_{2}\right),D_{2}^{*}\right)+b\log_{2}\sqrt{12D_{2}^{*}}\;\right]=bh\left(X_{2}\right). \end{array}$$

Equations (46)–(48) yield the following:

(49) $$\begin{array}{cc} \lim_{D\to 0} & \left[{\underline{\Delta }}_{m}\left(Q_{\mathrm{uni}}^{\left(1\right)},Q_{\mathrm{uni}}^{\left(2\right)},F_{s},D_{1}^{*},D_{2}^{*}\right)+\frac{a+b}{2}\log_{2}12D\right]\\ \; & \;=-\frac{a}{2}\log_{2}\frac{a}{\alpha (a+b)}-\frac{b}{2}\log_{2}\frac{b}{\left(1-\alpha )(a+b\right)}+ah\left(X_{1}\right)+bh\left(X_{2}\right)+c. \end{array}$$

Then, (45), (49), and Lemma 4 imply the following:

(50) $$\begin{array}{cc} \lim_{D\to 0} & \left[\Delta_{m}^{*}\left(D\right)+\frac{a+b}{2}\log_{2}12D\right]\\ \; & \;=-\frac{a}{2}\log_{2}\frac{a}{\alpha (a+b)}-\frac{b}{2}\log_{2}\frac{b}{\left(1-\alpha )(a+b\right)}+ah\left(X_{1}\right)+bh\left(X_{2}\right)+c. \end{array}$$

Then, we can directly obtain the following result:

(51) $$\begin{array}{c} \lim_{D\to 0}\frac{\Delta_{m}^{*}\left(D\right)}{\log_{2}D}=-\frac{a+b}{2}. \end{array}$$

This completes the proof. □

## 4. Asymptotically Optimal Solution for Single-Quantizer Compression Scheme

For the single-quantizer compression scheme, we develop an asymptotically optimal solution. Let $\beta =\frac{a}{a+b}$ and introduce two random variables ${\bar{X}}_{\alpha }$ and ${\bar{X}}_{\beta }$, with the pdfs ${\bar{f}}_{\alpha }\left(x\right)=\alpha f_{1}\left(x\right)+\left(1-\alpha \right)f_{2}\left(x\right)$ and ${\bar{f}}_{\beta }\left(x\right)=\beta f_{1}\left(x\right)+\left(1-\beta \right)f_{2}\left(x\right)$, respectively.

**Theorem 2.**  The pair $\left(Q_{w},F^{*}\right)$, consisting of the w-quantizer and the AoI-optimal encoder, forms an asymptotically optimal solution to the problem (14), as follows:

(52) $$\begin{array}{c} \lim_{D\to 0}\left[\Delta_{s}\left(Q_{w},F^{*}\right)-\Delta_{s}^{*}\left(D\right)\right]=0. \end{array}$$

*Under this solution, the asymptotic behavior of the optimal AoI satisfies the following:*

(53) $$\begin{array}{cc} \lim_{D\to 0}[\Delta_{s}^{*}\left(D\right) & +\frac{a+b}{2}\log_{2}12D]\\ \; & =\left(a+b\right)h\left({\bar{X}}_{\beta }\right)-\frac{a+b}{2}D({\bar{f}}_{\beta }\left(x\right)\left|\right|{\bar{f}}_{\alpha }\left(x\right))+c, \end{array}$$

*where $D({\bar{f}}_{\beta }\left(x\right)||{\bar{f}}_{\alpha }\left(x\right))=\int_{U}{\bar{f}}_{\beta }\left(x\right)\log_{2}\frac{{\bar{f}}_{\beta }\left(x\right)}{{\bar{f}}_{\alpha }\left(x\right)}dx$ is the relative entropy between ${\bar{f}}_{\beta }\left(x\right)$ and ${\bar{f}}_{\alpha }\left(x\right)$. Furthermore, we have the following:*

(54) $$\begin{array}{c} \lim_{D\to 0}\frac{\Delta_{s}^{*}\left(D\right)}{\log_{2}D}=-\frac{a+b}{2}. \end{array}$$

**Remark 6.**  *Theorem 2 reveals that the performance of the single-quantizer compression scheme also exhibits a strong dependence on the weight α and the ratio $\beta =\frac{a}{a+b}$. This scheme can essentially be viewed as a single-source compression problem, as implied in the proof below. Relative entropy $D({\bar{f}}_{\beta }\left(x\right)||{\bar{f}}_{\alpha }\left(x\right))$ characterizes the “mismatch" between the equivalent probability distribution in the objective function and that in the distortion constraint.*

**Figure 6:**
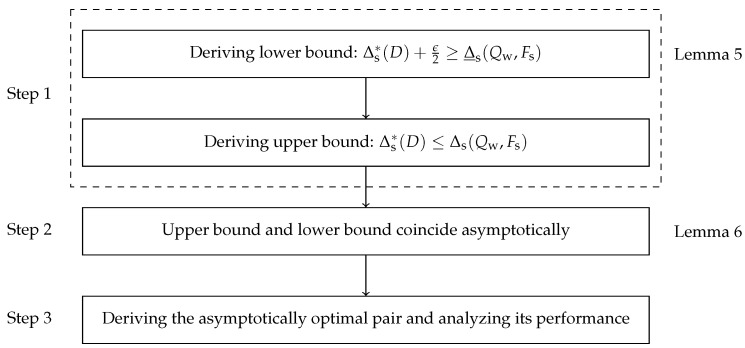
Proof flowchart of Theorem 2.

The proof of Theorem 2 proceeds as follows: First, for the single-quantizer compression scheme, we derive the upper and asymptotically lower bounds of the optimal AoI $\Delta_{s}^{*}\left(D\right)$. Then, we prove that the two bounds asymptotically coincide. Crucially, this is achieved by leveraging the Shannon encoder’s performance to approximate the solution of (14) in the high-resolution regime, thereby circumventing the need to solve the original nonlinear fractional optimization problem directly. Finally, we derive the asymptotically optimal pair and analyze its performance. The proof flowchart of Theorem 2 is shown in Figure 6.

**Proof.**  We divide the proof into the following three steps:

**Step 1.** Derive the upper and the asymptotically lower bounds of the optimal AoI $\Delta_{s}^{*}\left(D\right)$.

Let $w\left(x\right):=\frac{{\bar{f}}_{\alpha }\left(x\right)}{{\bar{f}}_{\beta }\left(x\right)}$. Then, the original WSMSE distortion metric in (11) is transformed into the following WMSE metric:

(55) $$\begin{array}{cc} D_{s}\left(X_{1},X_{2}\right)= & \alpha D\left(Q\left(X_{1}\right)\right)+\left(1-\alpha \right)D\left(Q\left(X_{2}\right)\right)\\ = & \sum_{j}\int_{a_{j-1}}^{a_{j}}{\left(x-c_{j}\right)}^{2}\left(\alpha f_{1}\left(x\right)dx+\left(1-\alpha \right)f_{2}\left(x\right)\right)dx \end{array}$$

(56) $$\begin{array}{cc} \;= & \sum_{j}\int_{a_{j-1}}^{a_{j}}{\left(x-c_{j}\right)}^{2}{\bar{f}}_{\alpha }\left(x\right)dx\\ \;= & \sum_{j}\int_{a_{j-1}}^{a_{j}}{\left(x-c_{j}\right)}^{2}w\left(x\right){\bar{f}}_{\beta }\left(x\right)dx=:D_{w}\left(Q\left({\bar{X}}_{\beta }\right)\right), \end{array}$$

By treating w(x) as a weight function, the original optimization problem (14) can be reformulated as follows:

(57) $$\begin{array}{cc} \; & \min_{\left\{Q\in Q,L\in L\right\}}\Delta_{s}\\ s.t. & \sum_{j}2^{-l_{j}}\leq 1,\;D_{w}\left(Q\left({\bar{X}}_{\beta }\right)\right)\leq D,\;l_{j}\in R^{+}. \end{array}$$

Then, we present an asymptotically lower bound of the optimal AoI $\Delta_{s}^{*}\left(D\right)$, as follows:

**Lemma 5.**  *For any ϵ>0, there exists a distortion $D^{\prime }>0$ such that $0<D<D^{\prime }$, the following inequality holds:*

(58) $$\begin{array}{c} \Delta_{s}^{*}\left(D\right)+\frac{\epsilon }{2}\geq {\underline{\Delta }}_{s}\left(Q_{w},F_{s}\right), \end{array}$$

*where*

(59) $$\begin{array}{c} {\underline{\Delta }}_{s}\left(Q_{w},F_{s}\right):=\left(a+b\right)H\left(Q_{w}\left({\bar{X}}_{\beta }\right)\right)+c. \end{array}$$

**Proof.**  By introducing the pmf $P_{\beta }=\left\{\beta p_{1}+\left(1-\beta \right)q_{1},\ldots ,\beta p_{j}+\left(1-\beta \right)q_{j},\ldots \right\}$, we obtain the following:

(60) $$\begin{array}{cc} \Delta_{s}\overset{\left(a\right)}{\geq } & aE_{P_{1}}\left[L\right]+bE_{P_{2}}\left[L\right]+c\\ = & a\sum_{j}p_{j}l_{j}+b\sum_{j}q_{j}l_{j}+c\\ = & \left(a+b\right)\sum_{j}\left(\frac{ap_{j}}{a+b}+\frac{bq_{j}}{a+b}\right)l_{j}+c\\ = & \left(a+b\right)\sum_{j}\left(\beta p_{j}+\left(1-\beta \right)q_{j}\right)l_{j}+c\\ = & \left(a+b\right)E_{P_{\beta }}\left[L\right]+c=:{\underline{\Delta }}_{s}, \end{array}$$

where (a) follows from Lemma 2.

For any ϵ>0, from the definition of infimum, there exists a pair $\left(Q^{\prime },F^{\prime }\right)$ satisfying $\Delta_{s}^{*}\left(D\right)+\frac{\epsilon }{4}>\Delta_{s}\left(Q^{\prime },F^{\prime }\right)$. In addition, from Lemma 1, we know that the *w*-quantizer is asymptotically optimal under the WMSE distortion. Therefore, there exists some $D^{\prime }>0$, such that $0<D<D^{\prime }$ implies $|H\left(Q_{w}\left({\bar{X}}_{\beta }\right)\right)-H\left(Q^{*}\left({\bar{X}}_{\beta }\right)\right)|<\frac{\epsilon }{4(a+b)}$. Then

(61) $$\begin{array}{cc} \Delta_{s}^{*}\left(D\right)+\frac{\epsilon }{2}\; & >\Delta_{s}\left(Q^{\prime },F^{\prime }\right)+\frac{\epsilon }{4}\\ \; & \geq \left(a+b\right)E_{P_{\beta }}\left[L\right]+c+\frac{\epsilon }{4}\\ \; & \overset{\left(a\right)}{\geq }\left(a+b\right)H\left(Q^{\prime }\left({\bar{X}}_{\beta }\right)\right)+c+\frac{\epsilon }{4}\\ \; & \geq \left(a+b\right)H\left(Q^{*}\left({\bar{X}}_{\beta }\right)\right)+c+\frac{\epsilon }{4}\\ \; & \geq \left(a+b\right)H\left(Q_{w}\left({\bar{X}}_{\beta }\right)\right)+c=:{\underline{\Delta }}_{s}\left(Q_{w},F_{s}\right), \end{array}$$

where (a) uses the fact that $E_{P_{\beta }}\left[L\right]\geq H\left(Q^{\prime }\left({\bar{X}}_{\beta }\right)\right)$ for a prefix-free code. This completes the proof. □

The AoI under the *w*-quantizer and the Shannon encoder provides an upper bound of the optimal AoI $\Delta_{s}^{*}\left(D\right)$. Then

(62) $$\begin{array}{c} {\underline{\Delta }}_{s}\left(Q_{w},F_{s}\right)<\Delta_{s}^{*}\left(D\right)+\frac{\epsilon }{2}\leq \Delta_{s}\left(Q_{w},F_{s}\right)+\frac{\epsilon }{2} \end{array}$$

**Step 2.** We prove that the two bounds asymptotically coincide.

**Lemma 6.**  *If the w-quantizer and the Shannon encoder are given, then we have the following:*

(63) $$\begin{array}{c} \lim_{D\to 0}\left[\Delta_{s}\left(Q_{w},F_{s}\right)-{\underline{\Delta }}_{s}\left(Q_{w},F_{s}\right)\right]=0. \end{array}$$

**Proof.**  See Appendix D. □

**Step 3.** Derive the asymptotically optimal pair and analyze its performance.

For any ϵ>0, there exists some $D^{\prime \prime }>0$, such that $0<D<D^{\prime \prime }$ implies $|\Delta_{s}\left(Q_{w},F_{s}\right)-{\underline{\Delta }}_{s}\left(Q_{w},F_{s}\right)|<\frac{\epsilon }{2}$. Thus, we have the following:

(64) $$\begin{array}{c} {\underline{\Delta }}_{s}\left(Q_{w},F_{s}\right)+\frac{\epsilon }{2}>\Delta_{s}\left(Q_{w},F_{s}\right)\geq \Delta_{s}\left(Q_{w},F^{*}\right). \end{array}$$

Letting $D_{0}=\min\left\{D^{\prime },D^{\prime \prime }\right\}$, for any *D* satisfying $0<D<D_{0}$, we have the following:

(65) $$\begin{array}{c} \Delta_{s}^{*}\left(D\right)+\epsilon >{\underline{\Delta }}_{s}\left(Q_{w},F_{s}\right)+\frac{\epsilon }{2}\geq \Delta_{s}\left(Q_{w},F^{*}\right). \end{array}$$

Since ϵ can be arbitrarily small, for sufficiently small *D*, the following results can be obtained:

(66) $$\begin{array}{c} \Delta_{s}^{*}\left(D\right)\geq \Delta_{s}\left(Q_{w},F^{*}\right). \end{array}$$

Thus, the *w*-quantizer and the AoI-optimal encoder are asymptotically optimal.

Next, we prove (53) and (54). We have the following:

(67) $$\begin{array}{cc} \; & \left|\Delta_{s}^{*}\left(D\right)+\frac{a+b}{2}\log_{2}12D-\left(a+b\right)h\left({\bar{X}}_{\beta }\right)-\frac{a+b}{2}E\left[\log_{2}\left(w\left({\bar{X}}_{\beta }\right)\right)\right]-c\right|\\ \leq & \left|\Delta_{s}\left(Q_{w},F_{s}\right)-{\underline{\Delta }}_{s}\left(Q_{w},F_{s}\right)\right|\\ \; & +|{\underline{\Delta }}_{s}\left(Q_{w},F_{s}\right)+\frac{a+b}{2}\log_{2}12D-\left(a+b\right)h\left({\bar{X}}_{\beta }\right)-\frac{a+b}{2}E\left[\log_{2}\left(w\left({\bar{X}}_{\beta }\right)\right)\right]-c|. \end{array}$$

By using Lemma 1, we obtain the following:

(68) $$\begin{array}{cc} \; & \lim_{D\to 0}\left[{\underline{\Delta }}_{s}\left(Q_{w},F_{s}\right)+\frac{a+b}{2}\log_{2}12D\right]\\ = & \lim_{D\to 0}\left[\left(a+b\right)H\left(Q_{w}\left({\bar{X}}_{\beta }\right)\right)+c+\frac{a+b}{2}\log_{2}12D\right]\\ = & \left(a+b\right)h\left({\bar{X}}_{\beta }\right)+\frac{a+b}{2}E\left[\log_{2}\left(w\left({\bar{X}}_{\beta }\right)\right)\right]+c\\ = & \left(a+b\right)h\left({\bar{X}}_{\beta }\right)+\frac{a+b}{2}\int_{U}{\bar{f}}_{\beta }\left(x\right)\log_{2}\frac{{\bar{f}}_{\alpha }\left(x\right)}{{\bar{f}}_{\beta }\left(x\right)}dx+c\\ = & \left(a+b\right)h\left({\bar{X}}_{\beta }\right)-\frac{a+b}{2}D({\bar{f}}_{\beta }\left(x\right)\left|\right|{\bar{f}}_{\alpha }\left(x\right))+c. \end{array}$$

Then, (67), (68) and Lemma 6 imply the following:

(69) $$\begin{array}{cc} \lim_{D\to 0}[\Delta_{s}^{*}\left(D\right) & +\frac{a+b}{2}\log_{2}12D]=\left(a+b\right)h\left({\bar{X}}_{\beta }\right)-\frac{a+b}{2}D({\bar{f}}_{\beta }\left(x\right)\left|\right|{\bar{f}}_{\alpha }\left(x\right))+c. \end{array}$$

From (69), the following result can be directly obtained:

(70) $$\begin{array}{c} \lim_{D\to 0}\frac{\Delta_{s}^{*}\left(D\right)}{\log_{2}D}=-\frac{a+b}{2}. \end{array}$$

This completes the proof. □

## 5. The Impact of System Parameters on AoI Performance

This section investigates how key parameters, such as arrival rates and weights of both sources, affect AoI performance in the high-resolution regime.

### 5.1. The Impact of Arrival Rates on AoI Performance

We first investigate how to optimally allocate arrival rates for two sources under a fixed total arrival rate $\lambda =\lambda_{1}+\lambda_{2}$ to minimize the system’s AoI performance. The main result is presented below.

**Proposition 1.**  In the high-resolution regime, when the total arrival rate λ is fixed, there exists a unique optimal rate allocation strategy $\left(\lambda_{1}^{*},\lambda -\lambda_{1}^{*}\right)$ that minimizes the system’s AoI performance for both the multi-quantizer compression scheme and the single-quantizer compression scheme.

**Proof.**  Similar to Lemma 2, the lower bound of AoI Δ is expressed as follows:

(71) $$\begin{array}{cc} \underline{\Delta }= & \left(1+\frac{\lambda_{1}}{\lambda -\lambda_{1}}+\frac{\lambda_{1}}{\lambda }\right)E\left[L_{1}\right]+\left(1+\frac{\lambda -\lambda_{1}}{\lambda_{1}}+\frac{\lambda -\lambda_{1}}{\lambda }\right)E\left[L_{2}\right]\\ \; & +\frac{\lambda_{1}^{2}+\lambda_{1}\left(\lambda -\lambda_{1}\right)+{\left(\lambda -\lambda_{1}\right)}^{2}}{\lambda_{1}\left(\lambda -\lambda_{1}\right)\lambda }. \end{array}$$

Taking the derivative of (71) with respect to $\lambda_{1}$ yields the following:

(72) $$\begin{array}{cc} \frac{d\underline{\Delta }}{d\lambda_{1}}= & \left(\frac{1}{\lambda }+\frac{\lambda }{{\left(\lambda -\lambda_{1}\right)}^{2}}\right)E\left[L_{1}\right]-\frac{\lambda^{2}+\lambda_{1}^{2}}{\lambda_{1}^{2}\lambda }E\left[L_{2}\right]-\frac{\lambda^{2}-2\lambda \lambda_{1}}{\lambda_{1}^{2}{\left(\lambda -\lambda_{1}\right)}^{2}}\\ = & \frac{\left(\lambda_{1}^{2}{\left(\lambda -\lambda_{1}\right)}^{2}+\lambda^{2}\lambda_{1}^{2}\right)E\left[L_{1}\right]-\left(\lambda^{2}+\lambda_{1}^{2}\right){\left(\lambda -\lambda_{1}\right)}^{2}E\left[L_{2}\right]-\lambda^{3}+2\lambda^{2}\lambda_{1}}{\lambda \lambda_{1}^{2}{\left(\lambda -\lambda_{1}\right)}^{2}}. \end{array}$$

Define

(73) $$\begin{array}{cc} G(\lambda_{1})= & \left(\lambda_{1}^{2}{\left(\lambda -\lambda_{1}\right)}^{2}+\lambda^{2}\lambda_{1}^{2}\right)E\left[L_{1}\right]-\left(\lambda^{2}+\lambda_{1}^{2}\right){\left(\lambda -\lambda_{1}\right)}^{2}E\left[L_{2}\right]-\lambda^{3}+2\lambda^{2}\lambda_{1}\\ = & \lambda_{1}^{2}{\left(\lambda -\lambda_{1}\right)}^{2}\left(E\left[L_{1}\right]-E\left[L_{2}\right]\right)+\lambda^{2}\left(\lambda_{1}^{2}E\left[L_{1}\right]-{\left(\lambda -\lambda_{1}\right)}^{2}E\left[L_{2}\right]\right)+2\lambda^{2}. \end{array}$$

Taking the derivative of (73) with respect to $\lambda_{1}$ results in the following:

(74) $$\begin{array}{cc} \frac{dG(\lambda_{1})}{d\lambda_{1}}= & \left(2\lambda_{1}{\left(\lambda -\lambda_{1}\right)}^{2}-2\left(\lambda -\lambda_{1}\right)\lambda_{1}^{2}\right)\left(E\left[L_{1}\right]-E\left[L_{2}\right]\right)\\ \; & +2\lambda^{2}\left(\lambda_{1}\left(E\left[L_{1}\right]-E\left[L_{2}\right]\right)+\lambda E\left[L_{2}\right]\right)+2\lambda^{2}. \end{array}$$

For the multi-quantizer scheme, when distortion D→0, we have

(75) $$\begin{array}{c} E\left[L_{1}\left(Q_{\mathrm{uni}}^{\left(1\right)},F_{s}\right)\right]-E\left[L_{2}\left(Q_{\mathrm{uni}}^{\left(2\right)},F_{s}\right)\right]\to h\left(X_{1}\right)-h\left(X_{2}\right)-\sqrt{\frac{a}{b}} \end{array}$$

and

(76) $$\begin{array}{c} E\left[L_{1}\left(Q_{\mathrm{uni}}^{\left(1\right)},F_{s}\right)\right]\to \infty ,\;E\left[L_{2}\left(Q_{\mathrm{uni}}^{\left(2\right)},F_{s}\right)\right]\to \infty . \end{array}$$

Thus, for sufficiently small *D*, we have

(77) $$\begin{array}{c} \frac{dG(\lambda_{1})}{d\lambda_{1}}>0. \end{array}$$

In the high-resolution regime, $G(\lambda_{1})$ monotonically increases with $\lambda_{1}$. Since G(λ)>0 and G(0)<0, the equation $G(\lambda_{1})=0$ has a unique solution, denoted by $\lambda_{1}^{*}$. Therefore, for the multi-quantizer scheme, there exists a unique optimal rate allocation strategy $\left(\lambda_{1}^{*},\lambda -\lambda_{1}^{*}\right)$ that minimizes the system’s AoI performance.

For the single-quantizer scheme, when distortion D→0, we have

(78) $$\begin{array}{c} E_{P_{1}}\left[L\left(Q_{w},F_{s}\right)\right]-E_{P_{2}}\left[L\left(Q_{w},F_{s}\right)\right]\to -\int_{U}f\left(x\right)\log_{2}\frac{{\bar{f}}_{\beta }\left(x\right)}{\sqrt{w(x)}}dx+\int_{U}f_{2}\left(x\right)\log_{2}\frac{{\bar{f}}_{\beta }\left(x\right)}{\sqrt{w(x)}}dx \end{array}$$

and

(79) $$\begin{array}{c} E_{P_{1}}\left[L\left(Q_{w},F_{s}\right)\right]\to \infty ,\;E_{P_{2}}\left[L\left(Q_{w},F_{s}\right)\right]\to \infty . \end{array}$$

For the single-quantizer scheme, by using a similar analysis, we also conclude that there exists a unique optimal rate allocation strategy that minimizes the system’s AoI performance in the high-resolution regime. This completes the proof. □

### 5.2. The Impact of Weights on AoI Performance

We now investigate the impact of both sources’ weights on AoI performance in the high-resolution regime for both compression schemes.

**Proposition 2.**  *For the multi-quantizer scheme, in the high-resolution regime, the optimal AoI performance is a concave function of weight α, uniquely achieving its maximum at α=β.*

**Proof.**  Define

(80) $$\begin{array}{cc} F_{1}\left(\alpha \right)= & -\frac{a}{2}\log_{2}\frac{a}{\alpha (a+b)}-\frac{b}{2}\log_{2}\frac{b}{\left(1-\alpha )(a+b\right)}+ah\left(X_{1}\right)+bh\left(X_{2}\right)+c. \end{array}$$

Taking the derivative of (80) with respect to α yields the following:

(81) $$\begin{array}{c} \frac{dF_{1}\left(\alpha \right)}{d\alpha }=\frac{a}{2\ln2}\frac{1}{\alpha }-\frac{b}{2\ln2}\frac{1}{1-\alpha }. \end{array}$$

Setting $\frac{dF_{1}\left(\alpha \right)}{d\alpha }=0$ results in the following:

(82) $$\begin{array}{c} \alpha =\beta . \end{array}$$

Taking the second-order derivative of (80) with respect to α yields the following:

(83) $$\begin{array}{c} \frac{d^{2}F_{1}\left(\alpha \right)}{d\alpha^{2}}=-\frac{a}{2\ln2}\frac{1}{\alpha^{2}}-\frac{b}{2\ln2}\frac{1}{{\left(1-\alpha \right)}^{2}}<0, \end{array}$$

establishing concavity with the unique maximum achieved at α=β. This completes the proof. □

**Proposition 3.**  *For the single-quantizer scheme, in the high-resolution regime, the optimal AoI performance is a concave function of weight α, uniquely achieving its maximum at α=β.*

**Proof.**  Define

(84) $$\begin{array}{cc} F_{2}\left(\alpha \right)= & \left(a+b\right)h\left({\bar{X}}_{\beta }\right)-\frac{a+b}{2}D({\bar{f}}_{\beta }\left(x\right)\left|\right|{\bar{f}}_{\alpha }\left(x\right))+c\\ = & \left(a+b\right)h\left({\bar{X}}_{\beta }\right)+\frac{a+b}{2}\int_{U}{\bar{f}}_{\beta }\left(x\right)\log_{2}\frac{{\bar{f}}_{\alpha }\left(x\right)}{{\bar{f}}_{\beta }\left(x\right)}dx+c. \end{array}$$

Taking the derivative of (84) with respect to α yields the following:

(85) $$\begin{array}{c} \frac{dF_{2}\left(\alpha \right)}{d\alpha }=\frac{a+b}{2\ln2}\int_{U}{\bar{f}}_{\beta }\left(x\right)\frac{f(x)-g(x)}{{\bar{f}}_{\alpha }\left(x\right)}dx. \end{array}$$

Setting $\frac{dF_{2}\left(\alpha \right)}{d\alpha }=0$ results in the following:

(86) $$\begin{array}{c} \alpha =\beta . \end{array}$$

Taking the second-order derivative of (84) with respect to α yields the following:

(87) $$\begin{array}{c} \frac{d^{2}F_{2}\left(\alpha \right)}{d\alpha^{2}}=-\frac{a+b}{2\ln2}\int_{U}{\bar{f}}_{\beta }\left(x\right)\frac{{\left(f\left(x\right)-g\left(x\right)\right)}^{2}}{{\bar{f}}_{\alpha }^{2}\left(x\right)}dx<0, \end{array}$$

establishing concavity with the unique maximum achieved at α=β. This completes the proof. □

## 6. Numerical Results

In this section, we present numerical results to evaluate the performance of the proposed solution. For sources 1 and 2, to satisfy the assumptions of pdfs, we truncate the pdfs $f_{1}\left(x\right)∼N\left(3,1\right)$ and $f_{2}\left(x\right)∼\exp\left(1\right)$ to the interval [0,6], respectively. We use “Upper bound1” and “Lower bound1” to represent the upper bound and asymptotically lower bound of the optimal performance for the multi-quantizer compression scheme, respectively. Moreover, we use “Upper bound2” and “Lower bound2” to represent the upper bound and asymptotically lower bound of the optimal performance for the single-quantizer compression scheme, respectively. In addition, we use “Fixed-length1” and “Fixed-length2” to represent the fixed-length encoding for the multi-quantizer compression scheme and the single-quantizer compression scheme, respectively.

**Figure 7:**
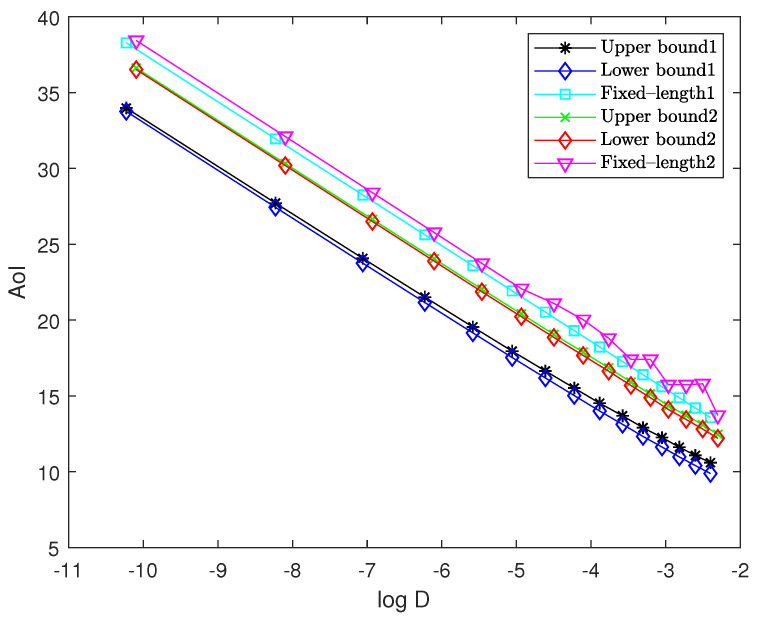
Performance comparison between upper bound, asymptotically lower bound, and corresponding fixed-length encoding scheme of optimal AoI versus log distortion for both schemes.

Figure 7 illustrates the upper bound and the asymptotically lower bound of the optimal AoI versus log distortion for both compression schemes. The arrival rates for sources 1 and 2 are set to $\lambda_{1}=3$ and $\lambda_{2}=1$, respectively. The weight is set to w=0.6. Through calculation, we derive the parameters $a=\frac{19}{4}$ and $b=\frac{19}{12}$. As the step size δ decreases from 1.5 to 0.1, the performance curve moves from the lower right to the upper left. For the multi-quantizer compression scheme, we implement uniform quantizers with cell sizes $\delta_{1}=\sqrt{\frac{a}{\alpha (a+b)}}\delta$ for source 1 and $\delta_{2}=\sqrt{\frac{b}{\left(1-\alpha )(a+b\right)}}\delta$ for source 2, each paired with their corresponding Shannon codes. The resulting AoI—plotted as the black curve—serves as the upper bound of the optimal AoI, while the asymptotically lower bound of the optimal AoI is plotted as the blue curve.

For the single-quantizer compression scheme, we employ the *w*-quantizer with its corresponding Shannon code. The resulting AoI—plotted as the green curve—is the upper bound of the optimal AoI, while the asymptotically lower bound is plotted as the red curve. We observe that the gaps between the upper and lower bounds for both schemes are remarkably small. As the quantization step size decreases, the gaps asymptotically approach zero, confirming Lemmas 4 and 6. In addition, four curves exhibit asymptotically linear behavior with the same slope of $-\frac{a+b}{2}=-\frac{19}{6}$, confirming Theorems 1 and 2. Furthermore, while maintaining the previously described quantizer structures, we evaluate the performance of fixed-length encoding for the multi-quantizer compression scheme (light blue curve) and the single-quantizer compression scheme (magenta curve). As we can observe, significant performance gaps exist between fixed-length encoding and the theoretical asymptotic optimum for both schemes. Additionally, the single-quantizer scheme (magenta curve) exhibits jitter at low bit rates. This jitter phenomenon stems from the design principle of the *w*-quantizer, which inserts about $\sqrt{w(x_{i})}$ quantization points within each interval on the basis of the uniform quantizer with cell size δ. At low bit rates (where δ is large), $w(x_{i})$ cannot be treated as a constant within each interval, leading to poor approximation performance. As δ decreases, this jitter effect gradually diminishes and eventually disappears when the quantization becomes sufficiently fine.

**Figure 8:**
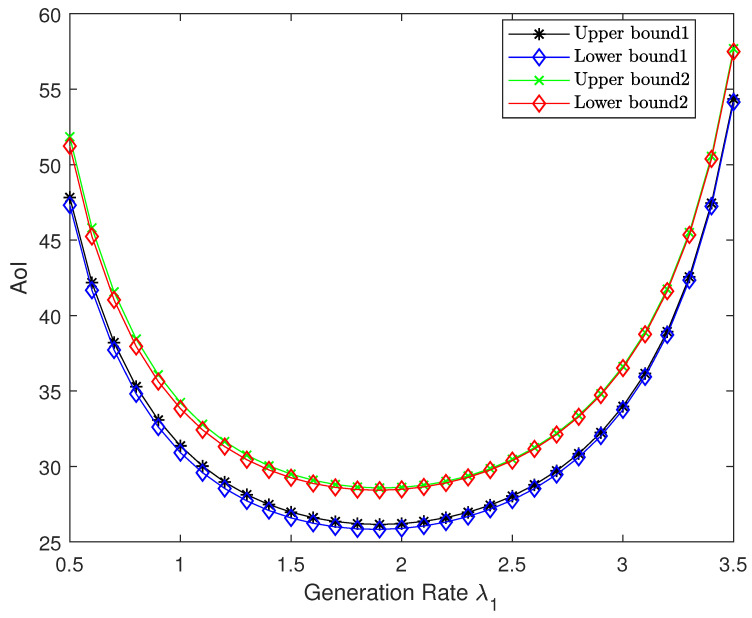
Performance comparison between upper bound and asymptotically lower bound with respect to source 1’s arrival rate $\lambda_{1}$ for both schemes.

Figure 8 illustrates the impact of source 1’s arrival rate $\lambda_{1}$ on the optimal AoI for both compression schemes in the high-resolution regime, with a fixed total arrival rate λ=4, weight w=0.6, and step size δ=0.1. We vary $\lambda_{1}$ from 0.5 to 3.5 in steps of 0.1 and plot the corresponding upper bounds and asymptotically lower bounds for both compression schemes. Notably, the gap between the upper and lower bound remains small for each scheme. In addition, the bounds for both compression schemes exhibit an initial monotonic decrease followed by a subsequent increase with respect to $\lambda_{1}$, with a unique minimum, confirming Proposition 1. This behavior can be explained through information freshness dynamics. As the arrival rate of either source becomes very small, the corresponding source experiences severely diminished update frequency, resulting in substantial age accumulation that dominates the system’s overall AoI performance. Specifically, when $\lambda_{1}$ is too small, source 1’s infrequent updates create an age bottleneck; conversely, when $\lambda_{1}$ approaches λ (making $\lambda_{2}$ small), source 2 becomes the freshness-limiting factor. This dependency creates the observed profile, with the optimal point occurring at a balanced rate allocation that avoids either extreme.

**Figure 9:**
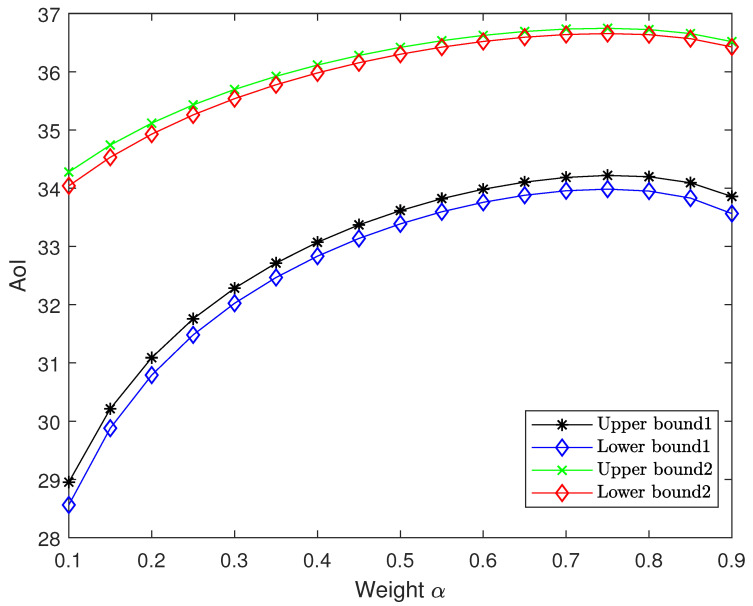
Performance comparison between upper bound and asymptotically lower bound of optimal AoI with respect to weight α for both schemes.

Figure 9 analyzes the effect of the weight α on the optimal AoI performance for both schemes in the high-resolution regime. The arrival rates for sources 1 and 2 are set to $\lambda_{1}=3$ and $\lambda_{2}=1$, respectively. The step size is set to δ=0.1. We vary α from 0.1 to 0.9 in steps of 0.05 and plot the upper and the asymptotically lower bounds. Our results reveal that the bounds exhibit concave behavior with respect to α, with $\alpha =\frac{a}{a+b}=0.75$ as the unique maximum, confirming Propositions 2 and 3.

Numerical simulations demonstrate that the multi-quantizer compression scheme, with its additional design flexibility, achieves better performance compared to the single-quantizer compression scheme. The multi-quantizer compression scheme can customize quantization cells for each source, while the single-quantizer scheme treats multiple sources as a single equivalent source, limiting its adaptability.

## 7. Conclusions

In this work, we consider a compression problem in a multi-source system to characterize the age–distortion tradeoff. We propose a multi-quantizer compression scheme and a single-quantizer compression scheme. For each scheme, we derive the asymptotically optimal solution and prove that the optimal AoI is asymptotically linear with respect to the log WSMSE, with the same slope determined by the sources’ arrival rates. Numerical simulations demonstrate that the multi-quantizer compression scheme, with its additional design flexibility, achieves better performance compared to the single-quantizer compression scheme. Furthermore, the proof of our results is based on an approximation technique to bypass a complex codeword length assignment problem. This method not only streamlines the theoretical analysis but also exhibits strong extensibility to other similar problems.

## Data Availability

The original contributions presented in this study are included in the article. Further inquiries can be directed to the corresponding author.

## Appendix A. Proof of Lemma 2

We analyze the following difference:

(A1) $$\begin{array}{cc} \Delta_{m}-{\underline{\Delta }}_{m}= & \frac{1}{\lambda_{1}}+\frac{1}{\lambda_{2}}-\frac{\lambda_{1}^{2}+\lambda_{1}\lambda_{2}+\lambda_{2}^{2}}{\lambda_{1}\lambda_{2}\left(\lambda_{1}+\lambda_{2}\right)}+\frac{\lambda_{1}E\left[L_{1}^{2}\right]+\lambda_{2}E\left[L_{2}^{2}\right]}{\lambda_{1}E\left[L_{1}\right]+\lambda_{2}E\left[L_{2}\right]+1}\\ \; & -\frac{\lambda_{1}}{\lambda_{1}+\lambda_{2}}E\left[L_{1}\right]-\frac{\lambda_{2}}{\lambda_{1}+\lambda_{2}}E\left[L_{2}\right]\\ = & \frac{\lambda_{1}^{2}\left(E\left[L_{1}^{2}\right]-E^{2}\left[L_{1}\right]\right)+\lambda_{2}^{2}\left(E\left[L_{2}^{2}\right]-E^{2}\left[L_{2}\right]\right)+1}{\left(\lambda_{1}E\left[L_{1}\right]+\lambda_{2}E\left[L_{2}\right]+1\right)\left(\lambda_{1}+\lambda_{2}\right)}\\ \; & +\frac{\left(\lambda_{1}\lambda_{2}\right)E\left[L_{1}^{2}\right]-2\left(\lambda_{1}\lambda_{2}\right)E\left[L_{1}\right]E\left[L_{2}\right]+\left(\lambda_{1}\lambda_{2}\right)E\left[L_{2}^{2}\right]}{\left(\lambda_{1}E\left[L_{1}\right]+\lambda_{2}E\left[L_{2}\right]+1\right)\left(\lambda_{1}+\lambda_{2}\right)}\\ \overset{\left(a\right)}{\geq } & \frac{\lambda_{1}^{2}\left(E\left[L_{1}^{2}\right]-E^{2}\left[L_{1}\right]\right)+\lambda_{2}^{2}\left(E\left[L_{2}^{2}\right]-E^{2}\left[L_{2}\right]\right)+\left(\lambda_{1}\lambda_{2}\right){\left(E\left[L_{1}\right]-E\left[L_{2}\right]\right)}^{2}+1}{\left(\lambda_{1}E\left[L_{1}\right]+\lambda_{2}E\left[L_{2}\right]+1\right)\left(\lambda_{1}+\lambda_{2}\right)}\geq 0, \end{array}$$

where (a) uses Jensen’s inequality. This completes the proof.

## Appendix B. Proof of Lemma 3

The distortion constraint specified in (8) gives rise to the following two cases:

**Case 1: Equality Constraint.** Consider the case where the given distortions $D_{1}$ and $D_{2}$ for sources 1 and 2 that satisfy the equality $\alpha D_{1}+\left(1-\alpha \right)D_{2}=D$. From the definition of infimum, we know that, for any ϵ>0, there exists a triplet $\left(Q_{1}^{\prime },Q_{2}^{\prime },F^{\prime }\right)$ satisfying $\Delta_{m}^{*}\left(D_{1},D_{2}\right)+\frac{1}{2}\epsilon >\Delta_{m}\left(Q_{1}^{\prime },Q_{2}^{\prime },F^{\prime },D_{1},D_{2}\right)$. Furthermore, due to the asymptotic optimality of the uniform quantizer for entropy-constrained quantization, we obtain that, for any ϵ>0, there exists some $D_{3}>0$ and $D_{4}>0$, such that $0<D_{1}<D_{3}$ and $0<D_{2}<D_{4}$ implies

(A2) $$\begin{array}{c} |H\left(Q_{\mathrm{uni}}^{\left(1\right)}\left(X_{1}\right),D_{1}\right)-H\left(Q_{1}^{*}\left(X_{1}\right),D_{1}\right)|<\frac{\epsilon }{4a}\\ |H\left(Q_{\mathrm{uni}}^{\left(2\right)}\left(X_{2}\right),D_{2}\right)-H\left(Q_{2}^{*}\left(X_{2}\right),D_{2}\right)|<\frac{\epsilon }{4b}. \end{array}$$

At the same time, there exists some $D_{5}>0$ and $D_{6}>0$, such that $0<D_{1}<D_{5}$ and $0<D_{2}<D_{6}$ implies the following:

(A3) $$\begin{array}{c} |H\left(Q_{\mathrm{uni}}^{\left(1\right)}\left(X_{1}\right),D_{1}\right)-\log_{2}\sqrt{12D_{1}}-h\left(X_{1}\right)|<\frac{\epsilon }{4a}\\ |H\left(Q_{\mathrm{uni}}^{\left(2\right)}\left(X_{2}\right),D_{2}\right)-\log_{2}\sqrt{12D_{2}}-h\left(X_{2}\right)|<\frac{\epsilon }{4b}. \end{array}$$

Letting $D_{0}=\min\left\{D_{3},D_{4},D_{5},D_{6}\right\}$, for any $D_{1}$, $D_{2}$ satisfying $0<D_{1}<D_{0}$ and $0<D_{2}<D_{0}$, we obtain the following:

(A4) $$\begin{array}{cc} \; & \Delta_{m}\left(Q_{1}^{\prime },Q_{2}^{\prime },F^{\prime },D_{1},D_{2}\right)+\epsilon \\ \geq & \Delta_{m}\left(Q_{1}^{\prime },Q_{2}^{\prime },F^{*},D_{1},D_{2}\right)+\epsilon \\ \overset{\left(a\right)}{\geq } & aE\left[L_{1}\right]+bE\left[L_{2}\right]+c+\epsilon \\ \geq & aH\left(Q_{1}^{\prime }\left(X_{1}\right),D_{1}\right)+bH\left(Q_{2}^{\prime }\left(X_{2}\right),D_{2}\right)+c+\epsilon \\ \overset{\left(b\right)}{\geq } & aH\left(Q_{1}^{*}\left(X_{1}\right),D_{1}\right)+bH\left(Q_{2}^{*}\left(X_{2}\right),D_{2}\right)+c+\epsilon \\ > & aH\left(Q_{\mathrm{uni}}^{\left(1\right)}\left(X_{1}\right),D_{1}\right)+bH\left(Q_{\mathrm{uni}}^{\left(2\right)}\left(X_{2}\right),D_{2}\right)+c+\frac{\epsilon }{2}\\ > & a\left(h\left(X_{1}\right)-\log_{2}\sqrt{12D_{1}}\right)+b\left(h\left(X_{2}\right)-\log_{2}\sqrt{12D_{2}}\right)+c\\ = & ah\left(X_{1}\right)+bh\left(X_{2}\right)+c-\frac{a}{2}\log_{2}12D_{1}-\frac{b}{2}\log_{2}12D_{2}, \end{array}$$

where (a) follows from Lemma 2, and (b) is due to the definition of the optimal quantizer. Let

(A5) $$\begin{array}{cc} F(D_{1})= & -\frac{a}{2}\log_{2}12D_{1}-\frac{b}{2}\log_{2}12\left(\frac{D-\alpha D_{1}}{1-\alpha }\right)+ah\left(X_{1}\right)+bh\left(X_{2}\right)+c. \end{array}$$

Taking the derivative of $F(D_{1})$ with respect to $D_{1}$ yields the following:

(A6) $$\begin{array}{c} F^{\prime }\left(D_{1}\right)=-\frac{a}{\left(2\ln2\right)D_{1}}+\frac{\alpha b}{\left(2\ln2\right)\left(D-\alpha D_{1}\right)}. \end{array}$$

Letting $F^{\prime }\left(D_{1}\right)=0$ results in the following:

(A7) $$\begin{array}{c} D_{1}^{*}=\frac{aD}{\alpha (a+b)}. \end{array}$$

Since $F^{\prime \prime }\left(D_{1}\right)>0$, the solution $D_{1}^{*}$ indeed represents the global minimum. Consequently, the optimal distortion allocation strategy between the two sources is given by $\left(D_{1}^{*},D_{2}^{*}\right)$, where $D_{1}^{*}=\frac{aD}{\alpha (a+b)}$ and $D_{2}^{*}=\frac{bD}{\left(1-\alpha )(a+b\right)}$. Then

(A8) $$\begin{array}{c} \Delta_{m}^{*}\left(D_{1},D_{2}\right)+\epsilon >aH\left(Q_{\mathrm{uni}}^{\left(1\right)}\left(X_{1}\right),D_{1}^{*}\right)+bH\left(Q_{\mathrm{uni}}^{\left(2\right)}\left(X_{2}\right),D_{2}^{*}\right)+c. \end{array}$$

Due to the arbitrary selection of distortions $D_{1}$ and $D_{2}$ satisfying the equality constraint $\alpha D_{1}+\left(1-\alpha \right)D_{2}=D$, then $\Delta_{m}^{*}\left(D\right)$ is asymptotically lower bounded by the following:

(A9) $$\begin{array}{c} \Delta_{m}^{*}\left(D\right)+\epsilon >aH\left(Q_{\mathrm{uni}}^{\left(1\right)}\left(X_{1}\right),D_{1}^{*}\right)+bH\left(Q_{\mathrm{uni}}^{\left(2\right)}\left(X_{2}\right),D_{2}^{*}\right)+c={\underline{\Delta }}_{m}\left(Q_{\mathrm{uni}}^{\left(1\right)},Q_{\mathrm{uni}}^{\left(2\right)},F_{s},D_{1}^{*},D_{2}^{*}\right). \end{array}$$

**Case 2: Inequality Constraint.** Consider the case where the given distortions $D_{1}^{\prime }$ and $D_{2}^{\prime }$ satisfy the inequality $\alpha D_{1}^{\prime }+\left(1-\alpha \right)D_{2}^{\prime }<D$. Then, there exists a slack variable γ>0 such that $\alpha D_{1}^{\prime }+\left(1-\alpha \right)D_{2}^{\prime }+\gamma =D$. In the high-resolution regime, we obtain the following:

(A10) $$\begin{array}{cc} \; & aH\left(Q_{\mathrm{uni}}^{\left(1\right)}\left(X_{1}\right),D_{1}^{\prime }\right)+bH\left(Q_{\mathrm{uni}}^{\left(2\right)}\left(X_{2}\right),D_{2}^{\prime }\right)\\ \geq & aH\left(Q_{\mathrm{uni}}^{\left(1\right)}\left(X_{1}\right),D_{1}^{\prime }\right)+bH\left(Q_{\mathrm{uni}}^{\left(2\right)}\left(X_{2}\right),D_{2}^{\prime }+\frac{\gamma }{1-\alpha }\right). \end{array}$$

Thus for the case of $\alpha D_{1}^{\prime }+\left(1-\alpha \right)D_{2}^{\prime }<D$, there always exists $D_{1}^{\prime }$ and $D_{2}^{\prime }+\frac{\gamma }{1-\alpha }$, which can achive a smaller value. Therefore, the optimal AoI can only be achieved when the equality constraint is satisfied. This completes the proof.

## Appendix C. Proof of Lemma 4

Define

(A11) $$\begin{array}{cc} \Phi_{m}:= & \frac{\lambda_{1}^{2}\left(E\left[L_{1}^{2}\right]-E^{2}\left[L_{1}\right]\right)+\lambda_{2}^{2}\left(E\left[L_{2}^{2}\right]-E^{2}\left[L_{2}\right]\right)+\left(\lambda_{1}\lambda_{2}\right){\left(E\left[L_{1}\right]-E\left[L_{2}\right]\right)}^{2}+1}{\left(\lambda_{1}E\left[L_{1}\right]+\lambda_{2}E\left[L_{2}\right]+1\right)\left(\lambda_{1}+\lambda_{2}\right)} \end{array}$$

and

(A12) $$\begin{array}{c} \Psi_{m}:=\Delta_{m}-{\underline{\Delta }}_{m}-\Phi_{m}. \end{array}$$

For sufficiently small distortion *D*, the optimal distortion allocation strategy $\left(D_{1}^{*},D_{2}^{*}\right)$ is as follows:

(A13) $$\begin{array}{c} D_{1}^{*}=\frac{aD}{\alpha (a+b)}\;D_{2}^{*}=\frac{bD}{\left(1-\alpha )(a+b\right)}. \end{array}$$

Since the uniform quantizer is asymptotically optimal, thus the cell sizes of two quantizers are $\delta_{1}=\sqrt{\frac{12aD}{\alpha (a+b)}}$ and $\delta_{2}=\sqrt{\frac{12bD}{\left(1-\alpha )(a+b\right)}}$ for sources 1 and 2, respectively. Then

(A14) $$\begin{array}{c} \delta_{1}=\sqrt{\frac{(1-\alpha )a}{\alpha b}}\delta_{2}. \end{array}$$

When D→0, then $\delta_{1}\to 0$ and $\delta_{2}\to 0$. For sufficiently small $\delta_{1}$ and $\delta_{2}$, the pdfs $f_{1}\left(x\right)$ and $f_{2}\left(x\right)$ can be approximated as constants within each cell. Under the uniform quantizer and the Shannon encoder, the second moments of the codeword lengths for the two sources are given by the following:

(A15) $$\begin{array}{cc} E[L_{1}^{2}\left(Q_{\mathrm{uni}}^{\left(1\right)},F_{s}\right)]= & \sum_{j_{1}}f_{1}\left(x_{j_{1}}\right)\delta_{1}{\left(\log_{2}f_{1}\left(x_{j_{1}}\right)\delta_{1}\right)}^{2}\\ = & \sum_{j_{1}}f_{1}\left(x_{j_{1}}\right)\delta_{1}\left(\log_{2}^{2}f_{1}\left(x_{j_{1}}\right)+2\log_{2}f_{1}\left(x_{j_{1}}\right)\log_{2}\delta_{1}\right)+\log_{2}^{2}\delta_{1} \end{array}$$

(A16) $$\begin{array}{cc} E[L_{2}^{2}\left(Q_{\mathrm{uni}}^{\left(2\right)},F_{s}\right)]= & \sum_{j_{2}}f_{2}\left(x_{j_{2}}\right)\delta_{2}{\left(\log_{2}f_{2}\left(x_{j_{2}}\right)\delta_{2}\right)}^{2}\\ = & \sum_{j_{2}}f_{2}\left(x_{j_{2}}\right)\delta_{2}\left(\log_{2}^{2}f_{2}\left(x_{j_{2}}\right)+2\log_{2}f_{2}\left(x_{j_{2}}\right)\log_{2}\delta_{2}\right)+\log_{2}^{2}\delta_{2}. \end{array}$$

The first moments of the codeword lengths for the two sources are as follows:

(A17) $$\begin{array}{cc} E[L_{1}\left(Q_{\mathrm{uni}}^{\left(1\right)},F_{s}\right)]= & -\sum_{j_{1}}f_{1}\left(x_{j_{1}}\right)\delta_{1}\left(\log_{2}f_{1}\left(x_{j_{1}}\right)\delta_{1}\right)\\ = & -\sum_{j_{1}}f_{1}\left(x_{j_{1}}\right)\delta_{1}\log_{2}f_{1}\left(x_{j_{1}}\right)-\log_{2}\delta_{1} \end{array}$$

(A18) $$\begin{array}{cc} E[L_{2}\left(Q_{\mathrm{uni}}^{\left(2\right)},F_{s}\right)]= & -\sum_{j_{2}}f_{2}\left(x_{j_{2}}\right)\delta_{2}\left(\log_{2}f_{2}\left(x_{j_{2}}\right)\delta_{2}\right)\\ = & -\sum_{j_{2}}f_{2}\left(x_{j_{2}}\right)\delta_{2}\log_{2}f_{2}\left(x_{j_{2}}\right)-\log_{2}\delta_{2}. \end{array}$$

Then, we have

(A19) $$\begin{array}{cc} \; & E\left[L_{1}^{2}\left(Q_{\mathrm{uni}}^{\left(1\right)},F_{s}\right)\right]-E^{2}\left[L_{1}\left(Q_{\mathrm{uni}}^{\left(1\right)},F_{s}\right)\right]\\ = & \sum_{j_{1}}f_{1}\left(x_{j_{1}}\right)\delta_{1}\log_{2}^{2}f_{1}\left(x_{j_{1}}\right)-{\left(\sum_{j_{1}}f_{1}\left(x_{j_{1}}\right)\delta_{1}\log_{2}f_{1}\left(x_{j_{1}}\right)\right)}^{2} \end{array}$$

and

(A20) $$\begin{array}{cc} \; & E\left[L_{2}^{2}\left(Q_{\mathrm{uni}}^{\left(2\right)},F_{s}\right)\right]-E^{2}\left[L_{2}\left(Q_{\mathrm{uni}}^{\left(2\right)},F_{s}\right)\right]\\ = & \sum_{j_{2}}f_{2}\left(x_{j_{2}}\right)\delta_{2}\log_{2}^{2}f_{2}\left(x_{j_{2}}\right)-{\left(\sum_{j_{2}}f_{2}\left(x_{j_{2}}\right)\delta_{2}\log_{2}f_{2}\left(x_{j_{2}}\right)\right)}^{2}. \end{array}$$

The difference between the first moments of the codeword lengths for the two sources is expressed as follows:

(A21) $$\begin{array}{cc} \; & E\left[L_{1}\left(Q_{\mathrm{uni}}^{\left(1\right)},F_{s}\right)\right]-E\left[L_{2}\left(Q_{\mathrm{uni}}^{\left(2\right)},F_{s}\right)\right]\\ = & -\sum_{j_{1}}f_{1}\left(x_{j_{1}}\right)\delta_{1}\log_{2}f_{1}\left(x_{j_{1}}\right)-\log_{2}\delta_{1}+\sum_{j_{2}}f_{2}\left(x_{j_{2}}\right)\delta_{2}\log_{2}f_{2}\left(x_{j_{2}}\right)+\log_{2}\delta_{2}\\ = & -\sum_{j_{1}}f_{1}\left(x_{j_{1}}\right)\delta_{1}\log_{2}f_{1}\left(x_{j_{1}}\right)-\log_{2}\sqrt{\frac{(1-\alpha )a}{\alpha b}}+\sum_{j_{2}}f_{2}\left(x_{j_{2}}\right)\delta_{2}\log_{2}f_{2}\left(x_{j_{2}}\right). \end{array}$$

When $\delta_{1}\to 0$, $\delta_{2}\to 0$, we have the following:

(A22) $$\begin{array}{cc} - & \sum_{j_{1}}f_{1}\left(x_{j_{1}}\right)\delta_{1}\log_{2}f_{1}\left(x_{j_{1}}\right)\to h\left(X_{1}\right) \end{array}$$

(A23) $$\begin{array}{cc} \; & \sum_{j_{1}}f_{1}\left(x_{j_{1}}\right)\delta_{1}\log_{2}^{2}f_{1}\left(x_{j_{1}}\right)\to \int_{U}f_{1}\left(x\right)\log_{2}^{2}f_{1}\left(x\right)dx \end{array}$$

(A24) $$\begin{array}{cc} - & \sum_{j_{2}}f_{2}\left(x_{j_{2}}\right)\delta_{2}\log_{2}f_{2}\left(x_{j_{2}}\right)\to h\left(X_{2}\right) \end{array}$$

(A25) $$\begin{array}{cc} \; & \sum_{j_{2}}f_{2}\left(x_{j_{2}}\right)\delta_{2}\log_{2}^{2}f_{2}\left(x_{j_{2}}\right)\to \int_{U}f_{2}\left(x\right)\log_{2}^{2}f_{2}\left(x\right)dx. \end{array}$$

Thus, we obtain the following:

(A26) $$\begin{array}{c} E\left[L_{1}\left(Q_{\mathrm{uni}}^{\left(1\right)},F_{s}\right)\right]-E\left[L_{2}\left(Q_{\mathrm{uni}}^{\left(2\right)},F_{s}\right)\right]\to h\left(X_{1}\right)-h\left(X_{2}\right)-\log_{2}\sqrt{\frac{(1-\alpha )a}{\alpha b}}. \end{array}$$

We can further derive the following:

(A27) $$\begin{array}{cc} \; & \lim_{D\to 0}\Phi_{m}\left(Q_{\mathrm{uni}}^{\left(1\right)},Q_{\mathrm{uni}}^{\left(2\right)},F_{s},D_{1}^{*},D_{2}^{*}\right)\\ = & \lim_{D\to 0}\frac{\lambda_{1}^{2}\left(E\left[L_{1}^{2}\left(Q_{\mathrm{uni}}^{\left(1\right)},F_{s}\right)\right]-E^{2}\left[L_{1}\left(Q_{\mathrm{uni}}^{\left(1\right)},F_{s}\right)\right]\right)}{\left(\lambda_{1}E\left[L_{1}\left(Q_{\mathrm{uni}}^{\left(1\right)},F_{s}\right)\right]+\lambda_{2}E\left[L_{2}\left(Q_{\mathrm{uni}}^{\left(2\right)},F_{s}\right)\right]+1\right)\left(\lambda_{1}+\lambda_{2}\right)}\\ \; & \;+\frac{\lambda_{2}^{2}\left(E\left[L_{2}^{2}\left(Q_{\mathrm{uni}},F_{s}\right)\right]-E^{2}\left[L_{2}\left(Q_{\mathrm{uni}}^{\left(2\right)},F_{s}\right)\right]\right)}{\left(\lambda_{1}E\left[L_{1}\left(Q_{\mathrm{uni}}^{\left(1\right)},F_{s}\right)\right]+\lambda_{2}E\left[L_{2}\left(Q_{\mathrm{uni}}^{\left(2\right)},F_{s}\right)\right]+1\right)\left(\lambda_{1}+\lambda_{2}\right)}\\ \; & \;+\frac{\left(\lambda_{1}\lambda_{2}\right){\left(E\left[L_{1}\left(Q_{\mathrm{uni}}^{\left(1\right)},F_{s}\right)\right]-E\left[L_{2}\left(Q_{\mathrm{uni}}^{\left(2\right)},F_{s}\right)\right]\right)}^{2}+1}{\left(\lambda_{1}E\left[L_{1}\left(Q_{\mathrm{uni}}^{\left(1\right)},F_{s}\right)\right]+\lambda_{2}E\left[L_{2}\left(Q_{\mathrm{uni}}^{\left(2\right)},F_{s}\right)\right]+1\right)\left(\lambda_{1}+\lambda_{2}\right)}\\ = & 0 \end{array}$$

and

(A28) $$\begin{array}{cc} \; & \lim_{D\to 0}\Psi_{m}\left(Q_{\mathrm{uni}}^{\left(1\right)},Q_{\mathrm{uni}}^{\left(2\right)},F_{s},D_{1}^{*},D_{2}^{*}\right)\\ = & \lim_{D\to 0}\frac{\left(\lambda_{1}\lambda_{2}\right)\left(E\left[L_{1}^{2}\left(Q_{\mathrm{uni}}^{\left(1\right)},F_{s}\right)\right]-E^{2}\left[L_{1}\left(Q_{\mathrm{uni}}^{\left(1\right)},F_{s}\right)\right]\right)}{\left(\lambda_{1}E\left[L_{1}\left(Q_{\mathrm{uni}}^{\left(1\right)},F_{s}\right)\right]+\lambda_{2}E\left[L_{2}\left(Q_{\mathrm{uni}}^{\left(2\right)},F_{s}\right)\right]+1\right)\left(\lambda_{1}+\lambda_{2}\right)}\\ \; & \;+\frac{\left(\lambda_{1}\lambda_{2}\right)\left(E\left[L_{2}^{2}\left(Q_{\mathrm{uni}}^{\left(2\right)},F_{s}\right)\right]-E^{2}\left[L_{2}\left(Q_{\mathrm{uni}}^{\left(2\right)},F_{s}\right)\right]\right)}{\left(\lambda_{1}E\left[L_{1}\left(Q_{\mathrm{uni}}^{\left(1\right)},F_{s}\right)\right]+\lambda_{2}E\left[L_{2}\left(Q_{\mathrm{uni}}^{\left(2\right)},F_{s}\right)\right]+1\right)\left(\lambda_{1}+\lambda_{2}\right)}\\ = & 0. \end{array}$$

Equations (A27) and (A28) yield the following:

(A29) $$\begin{array}{c} \lim_{D\to 0}\left[\Delta_{m}\left(Q_{\mathrm{uni}}^{\left(1\right)},Q_{\mathrm{uni}}^{\left(2\right)},F_{s},D_{1}^{*},D_{2}^{*}\right)-{\underline{\Delta }}_{m}\left(Q_{\mathrm{uni}}^{\left(1\right)},Q_{\mathrm{uni}}^{\left(2\right)},F_{s},D_{1}^{*},D_{2}^{*}\right)\right]=0. \end{array}$$

This completes the proof.

## Appendix D. Proof of Lemma 6

For the symbol ${\bar{X}}_{\beta }$, according to the construction of the *w*-quantizer, the occurrence probability of the *k*th cell in the *j*th interval is given by $\beta p_{j_{k}}+\left(1-\beta \right)q_{j_{k}}$, with the weight $w(x_{j_{k}})$. For a sufficiently small step size δ, both the pdf ${\bar{f}}_{\beta }\left(x\right)$ and the weight function w(x) can be approximated as constants ${\bar{f}}_{\beta }\left(x_{j}\right)$ and $w(x_{j})$ within each interval. We use the Shannon encoder, and the codeword length assigned to the *k*th cell in the *j*th interval is as follows:

(A30) $$\begin{array}{c} l_{j_{k}}=-\log_{2}\left(\beta p_{j_{k}}+\left(1-\beta \right)q_{j_{k}}\right)=-\log_{2}\frac{{\bar{f}}_{\beta }\left(x_{j_{k}}\right)\delta }{\sqrt{w(x_{j_{k}})}}. \end{array}$$

The second moment of the codeword lengths for source 1 is as follows:

(A31) $$\begin{array}{cc} E_{P_{1}}\left[L^{2}\left(Q_{w},F_{s}\right)\right]= & \sum_{j_{k}}f_{1}\left(x_{j_{k}}\right)\frac{\delta }{\sqrt{w(x_{j_{k}})}}\log_{2}^{2}\left({\bar{f}}_{\beta }\left(x_{j_{k}}\right)\frac{\delta }{\sqrt{w(x_{j_{k}})}}\right)\\ = & \sum_{j}f_{1}\left(x_{j}\right)\delta \log_{2}^{2}\left(\frac{{\bar{f}}_{\beta }\left(x_{j}\right)}{\sqrt{w(x_{j})}}\delta \right)\\ = & \sum_{j}f_{1}\left(x_{j}\right)\delta \log_{2}^{2}\frac{{\bar{f}}_{\beta }\left(x_{j}\right)}{\sqrt{w(x_{j})}}+2\sum_{j}f_{1}\left(x_{j}\right)\delta \left(\log_{2}\frac{{\bar{f}}_{\beta }\left(x_{j}\right)}{\sqrt{w(x_{j})}}\right)\log_{2}\delta +\log_{2}^{2}\delta . \end{array}$$

The first moment of the codeword lengths for source 1 is as follows:

(A32) $$\begin{array}{cc} E_{P_{1}}\left[L\left(Q_{w},F_{s}\right)\right]= & -\sum_{j_{k}}f_{1}\left(x_{j_{k}}\right)\frac{\delta }{\sqrt{w(x_{j_{k}})}}\log_{2}\left({\bar{f}}_{\beta }\left(x_{j_{k}}\right)\frac{\delta }{\sqrt{w(x_{j_{k}})}}\right)\\ = & -\sum_{j}f_{1}\left(x_{j}\right)\delta \log_{2}\left({\bar{f}}_{\beta }\left(x_{j}\right)\frac{\delta }{\sqrt{w(x_{j})}}\right)\\ = & -\sum_{j}f_{1}\left(x_{j}\right)\delta \log_{2}\left(\frac{{\bar{f}}_{\beta }\left(x_{j}\right)}{\sqrt{w(x_{j})}}\right)-\log_{2}\delta . \end{array}$$

When δ→0, we have the following:

(A33) $$\begin{array}{cc} \; & \sum_{j}f_{1}\left(x_{j}\right)\delta \log_{2}\frac{{\bar{f}}_{\beta }\left(x_{j}\right)}{\sqrt{w(x_{j})}}⟶\int_{U}f_{1}\left(x\right)\log_{2}\frac{{\bar{f}}_{\beta }\left(x\right)}{\sqrt{w(x)}}dx \end{array}$$

(A34) $$\begin{array}{cc} \; & \sum_{j}f_{1}\left(x_{j}\right)\delta \log_{2}^{2}\frac{{\bar{f}}_{\beta }\left(x_{j}\right)}{\sqrt{w(x_{j})}}⟶\int_{U}f_{1}\left(x\right)\log_{2}^{2}\frac{{\bar{f}}_{\beta }\left(x\right)}{\sqrt{w(x)}}dx. \end{array}$$

Then, we can obtain the following:

(A35) $$\begin{array}{cc} \; & E_{P_{1}}\left[L^{2}\left(Q_{w},F_{s}\right)\right]-E_{P_{1}}^{2}\left[L\left(Q_{w},F_{s}\right)\right]\\ = & \sum_{j}f_{1}\left(x_{j}\right)\delta \log_{2}^{2}\frac{{\bar{f}}_{\beta }\left(x_{j}\right)}{\sqrt{w(x_{j})}}-{\left(\sum_{j}f_{1}\left(x_{j}\right)\delta \log_{2}\frac{{\bar{f}}_{\beta }\left(x_{j}\right)}{\sqrt{w(x_{j})}}\right)}^{2} \end{array}$$

When δ→0, we derive the following:

(A36) $$\begin{array}{cc} \; & E_{P_{1}}\left[L^{2}\left(Q_{w},F_{s}\right)\right]-E_{P_{1}}^{2}\left[L\left(Q_{w},F_{s}\right)\right]\\ ⟶ & \int_{U}f_{1}\left(x\right)\log_{2}^{2}\frac{{\bar{f}}_{\beta }\left(x\right)}{\sqrt{w(x)}}dx-{\left(\int_{U}f_{1}\left(x\right)\log_{2}\frac{{\bar{f}}_{\beta }\left(x\right)}{\sqrt{w(x)}}dx\right)}^{2}. \end{array}$$

An analogous result holds for Source 2. When δ→0, we have

(A37) $$\begin{array}{cc} \; & E_{P_{2}}\left[L^{2}\left(Q_{w},F_{s}\right)\right]-E_{P_{2}}^{2}\left[L\left(Q_{w},F_{s}\right)\right]\\ ⟶ & \int_{U}f_{2}\left(x\right)\log_{2}^{2}\frac{{\bar{f}}_{\beta }\left(x\right)}{\sqrt{w(x)}}dx-{\left(\int_{U}f_{2}\left(x\right)\log_{2}\frac{{\bar{f}}_{\beta }\left(x\right)}{\sqrt{w(x)}}dx\right)}^{2}. \end{array}$$

The difference in the first moments of the codeword lengths between the two sources is expressed as follows:

(A38) $$\begin{array}{cc} \; & E_{P_{1}}\left[L\left(Q_{w},F_{s}\right)\right]-E_{P_{2}}\left[L\left(Q_{w},F_{s}\right)\right]\\ = & -\sum_{j}f_{1}\left(x_{j}\right)\delta \log_{2}\left({\bar{f}}_{\beta }\left(x_{j}\right)\frac{\delta }{\sqrt{w(x_{j})}}\right)+\sum_{j}f_{2}\left(x_{j}\right)\delta \log_{2}\left({\bar{f}}_{\beta }\left(x_{j}\right)\frac{\delta }{\sqrt{w(x_{j})}}\right)\\ = & -\sum_{j}f_{1}\left(x_{j}\right)\delta \log_{2}\left(\frac{{\bar{f}}_{\beta }\left(x_{j}\right)}{\sqrt{w(x_{j})}}\right)+\sum_{j}f_{2}\left(x_{j}\right)\delta \log_{2}\left(\frac{{\bar{f}}_{\beta }\left(x_{j}\right)}{\sqrt{w(x_{j})}}\right) \end{array}$$

when δ→0, we have the following:

(A39) $$\begin{array}{cc} \; & E_{P_{1}}\left[L\left(Q_{w},F_{s}\right)\right]-E_{P_{2}}\left[L\left(Q_{w},F_{s}\right)\right]\\ ⟶ & -\int_{U}f_{1}\left(x\right)\log_{2}\frac{{\bar{f}}_{\beta }\left(x\right)}{\sqrt{w(x)}}dx+\int_{U}f_{2}\left(x\right)\log_{2}\frac{{\bar{f}}_{\beta }\left(x\right)}{\sqrt{w(x)}}dx. \end{array}$$

Let

(A40) $$\begin{array}{cc} \Phi_{s}:=\; & \frac{\lambda_{1}^{2}\left(E_{P_{1}}\left[L^{2}\right]-E_{P_{1}}^{2}\left[L\right]\right)+\lambda_{2}^{2}\left(E_{P_{2}}\left[L^{2}\right]-E_{P_{2}}^{2}\left[L\right]\right)}{\left(\lambda_{1}E_{P_{1}}\left[L\right]+\lambda_{2}E_{P_{2}}\left[L\right]+1\right)\left(\lambda_{1}+\lambda_{2}\right)}\\ \; & \;+\frac{\left(\lambda_{1}\lambda_{2}\right){\left(E_{P_{1}}\left[L\right]-E_{P_{2}}\left[L\right]\right)}^{2}+1}{\left(\lambda_{1}E_{P_{1}}\left[L\right]+\lambda_{2}E_{P_{2}}\left[L\right]+1\right)\left(\lambda_{1}+\lambda_{2}\right)} \end{array}$$

and

(A41) $$\begin{array}{c} \Psi_{s}:=\Delta_{s}-{\underline{\Delta }}_{s}-\Phi_{s}. \end{array}$$

When D→0, we have δ→0. Then, we obtain the following:

(A42) $$\begin{array}{cc} \; & \lim_{D\to 0}\Phi_{s}\left(Q_{w},F_{s}\right)\\ = & \lim_{D\to 0}\frac{\lambda_{1}^{2}\left(E_{P_{1}}\left[L^{2}\left(Q_{w},F_{s}\right)\right]-E_{P_{1}}^{2}\left[L\left(Q_{w},F_{s}\right)\right]\right)}{\left(\lambda_{1}E_{P_{1}}\left[L\left(Q_{w},F_{s}\right)\right]+\lambda_{2}E_{P_{2}}\left[L\left(Q_{w},F_{s}\right)\right]+1\right)\left(\lambda_{1}+\lambda_{2}\right)}\\ \; & \;+\frac{\lambda_{2}^{2}\left(E_{P_{2}}\left[L^{2}\left(Q_{w},F_{s}\right)\right]-E_{P_{2}}^{2}\left[L\left(Q_{w},F_{s}\right)\right]\right)}{\left(\lambda_{1}E_{P_{1}}\left[L\left(Q_{w},F_{s}\right)\right]+\lambda_{2}E_{P_{2}}\left[L\left(Q_{w},F_{s}\right)\right]+1\right)\left(\lambda_{1}+\lambda_{2}\right)}\\ \; & \;+\frac{\left(\lambda_{1}\lambda_{2}\right){\left(E_{P_{1}}\left[L\left(Q_{w},F_{s}\right)\right]-E_{P_{2}}\left[L\left(Q_{w},F_{s}\right)\right]\right)}^{2}+1}{\left(\lambda_{1}E_{P_{1}}\left[L\left(Q_{w},F_{s}\right)\right]+\lambda_{2}E_{P_{2}}\left[L\left(Q_{w},F_{s}\right)\right]+1\right)\left(\lambda_{1}+\lambda_{2}\right)}\\ = & 0 \end{array}$$

and

(A43) $$\begin{array}{cc} \; & \lim_{D\to 0}\Psi_{s}\left(Q_{w},F_{s}\right)\\ = & \lim_{D\to 0}\frac{\left(\lambda_{1}\lambda_{2}\right)\left(E_{P_{1}}\left[L^{2}\left(Q_{w},F_{s}\right)\right]-E_{P_{1}}^{2}\left[L\left(Q_{w},F_{s}\right)\right]\right)}{\left(\lambda_{1}E_{P_{1}}\left[L\left(Q_{w},F_{s}\right)\right]+\lambda_{2}E_{P_{2}}\left[L\left(Q_{w},F_{s}\right)\right]+1\right)\left(\lambda_{1}+\lambda_{2}\right)}\\ \; & \;+\frac{\left(\lambda_{1}\lambda_{2}\right)\left(E_{P_{2}}\left[L{\left(Q_{w},F_{s}\right)}^{2}\right]-E_{P_{2}}^{2}\left[L\left(Q_{w},F_{s}\right)\right]\right)}{\left(\lambda_{1}E_{P_{1}}\left[L\left(Q_{w},F_{s}\right)\right]+\lambda_{2}E_{P_{2}}\left[L\left(Q_{w},F_{s}\right)\right]+1\right)\left(\lambda_{1}+\lambda_{2}\right)}\\ = & 0. \end{array}$$

Thus, we have

(A44) $$\begin{array}{c} \lim_{D\to 0}\left[\Delta_{s}\left(Q_{w},F_{s}\right)-{\underline{\Delta }}_{s}\left(Q_{w},F_{s}\right)\right]=0. \end{array}$$

This completes the proof.
